# Virus-specific CD8 T cells rapidly populate and persist in skull bone marrow after brain infection

**DOI:** 10.21203/rs.3.rs-9890268/v1

**Published:** 2026-06-03

**Authors:** Asma Hassani, Fang Jin, Michael D Forston, Armeta Hadjimirzaei, Cody L Lewis, Carley A Owens, Javonte S Thelwell, Marina Seady, Mark A Maynes, Zichen Tian, Michael J Hansen, Katayoun Ayasoufi, Aaron J Johnson

**Affiliations:** 1 Department of Immunology, Mayo Clinic, Rochester, MN 55905 USA; 2 Graduate School of Biomedical Sciences, Mayo Clinic, Rochester, MN 55905 USA; 3 Department of Molecular Pharmacology and Experimental Therapeutics, Mayo Clinic, Rochester, MN 55905 USA; 4 Department of Biochemistry and Molecular Biology, Mayo Clinic, Rochester, MN 55905 USA; 5 Department of Neuroscience, Mayo Clinic, Rochester, MN 55905 USA; 6 Department of Neurosurgery, Duke University, Durham, NC 27710 USA; 7 Department of Molecular Medicine, Mayo Clinic, Rochester, MN 55905 USA; 8 Department of Neurology, Mayo Clinic, Rochester, MN 55905 USA

## Abstract

Skull bone marrow (BM) is a recently identified site for immune activation responsive to CNS insults. The extent to which adaptive immunity is generated through skull BM is poorly understood. In this study, we evaluated how skull BM supports the early expansion of antiviral CD8 T cells during acute neurotropic virus infection. Virus antigen-specific CD8 T cells were rapidly identified in skull BM at a rate comparable to lymphoid compartments during acute Theiler’s murine encephalomyelitis viral (TMEV) infection. We therefore employed MHC class I conditional knockout mice and demonstrated the necessity of CD11c+ antigen-presenting cells (APCs) for the generation of various subsets of TMEV-specific CD8 T cells in skull BM. Importantly, the early expansion of skull BM-derived antiviral T cells was impeded by FTY720 treatment and restored upon shared circulation with a mouse exhibiting intact antigen presentation by CD11c+ APCs. Additionally, TMEV-specific CD8 T cells were retained in skull BM during both chronic CNS infection and long after infection has been resolved. Overall, we determined that skull BM responds to acute neurotropic viral infection through the rapid recruitment of virus-specific CD8 T cells primed in the periphery before serving as a reservoir for immunologic memory.

## Introduction

Owing to its proximity to the brain and the recent line of pioneering work revealing osseous channels protruding out of the inner skull cortex and stretching into the meninges, skull bone marrow (BM) has emerged as a critical anatomic site for immune responses reactive to central nervous system (CNS) insults. Skull BM displays an exceptional level of communication with the meninges and the brain, unlike other bone marrow compartments in the body^1^. This is supported by multiple recent observations demonstrating skull BM as a reservoir of monocytes and neutrophils that relocate to the meninges through vascularized bridges that facilitate bidirectional movements^2–6^. Skull BM was found to dispatch populations of neutrophils to the ischemic brain via transit through the meninges, a process triggered by discrete signals from meningeal mast cells^7^. In respect to adaptive immunity, the bulk of meningeal B cells were found to originate from skull BM^8,9^. Skull BM has also been shown to accumulate discrete subsets of CD8 T cells at steady state, offering a suitable environment for antigen-dependent interactions, primarily between central memory CD8 T cells and antigen-laden dendritic cells^10,11^.

Skull BM exhibits a distinctive proteomic and transcriptomic profile from other hematopoietic compartments^1^ and is highly responsive to brain injuries. Following intracerebral hemorrhage, skull BM was found to promote the release of repair-inducing anti-inflammatory monocytes. Similarly, skull BM-derived monocytes mobilized selectively to rims of brain lesions displaying an anti-inflammatory phenotype in experimental traumatic brain injury^12^. Skull BM was also found to be sensitive to cerebrospinal fluid (CSF) signals stemming from the tumor microenvironment, resulting in excessive myelopoiesis and preferential differentiation of CD4 T cells into immunosuppressive regulatory subsets in a murine model of pediatric ependymoma^13^. Furthermore, regions of skull BM associated with the cribriform plate within the nasal cavity are in close proximity to CSF-filled perineural spaces surrounding the olfactory nerve. This latter BM site was identified to be vulnerable to brain-invading bacteria, accessible to CSF-derived cues and displayed extensive myelopoiesis following neuroinflammation^14^. These studies underscore the role of skull BM in myelopoiesis, specifically the release of monocytes, neutrophils, and B cells in response to brain injury or infection. Nevertheless, the generation of adaptive immune responses remains centered on the capacity of cervical lymph nodes to receive drainage of CNS antigens to initiate antigen-specific T cell responses^15^. However, the extent skull BM supports the development of antiviral CD8 T cells in response to neurotropic viral infections remains unknown.

In this study, we sought to determine how skull BM contributes to antiviral CD8 T cell responses to neurotropic viral infections employing the Theiler’s murine encephalomyelitis virus (TMEV) infection model in C57BL/6 mice. TMEV, is a non-enveloped RNA virus of the *Picornaviridae* family, producing different pathologies in different mice strains. TMEV brain infection results in acute encephalomyelitis followed by effective recovery, with no evidence of viral persistence in C57BL/6 mice possessing H-2b haplotype^16–18^. VP2_121–130_ (FHAGSLLVFM) is the immunodominant TMEV epitope, presented by the MHC class I molecule, H-2D^b^, in C57BL/6 mice for which successful viral clearance by antiviral cytotoxic CD8 T cell responses is achieved^19–21^. CD8 T cells specific to H-2D^b^-restricted VP2_121–130_, and less frequently to H-2K^b^-restricted viral epitopes, are generated in several secondary lymphoid organs and infiltrate the infected brain^22^. Several reports have shown that virus-specific T cells develop in different BM compartments in humans and mice^23–26^. To this end, we hypothesized that acute brain infection with TMEV triggers rapid expansion and long-term persistence of virus antigen-specific CD8 T cells in skull BM. In this work, we provide compelling evidence for the early expansion of virus-specific CD8 T cells in skull BM, activated by discrete antigen-presenting cells (APCs) following TMEV infection. We further demonstrate that skull BM retains virus-specific CD8 T cells long after the resolution of infection. Leveraging our established transgenic mice with conditional deletion of single MHC class I molecule on APCs, pharmacological inhibition of immune cell trafficking, and parabiosis, we illustrate that virus-specific CD8 T cells in skull BM are primed in the periphery and rapidly migrate to skull BM during acute neurotropic virus infection.

## Results

### TMEV RNA gains access to skull BM during acute brain infection

**Fig. 1.**
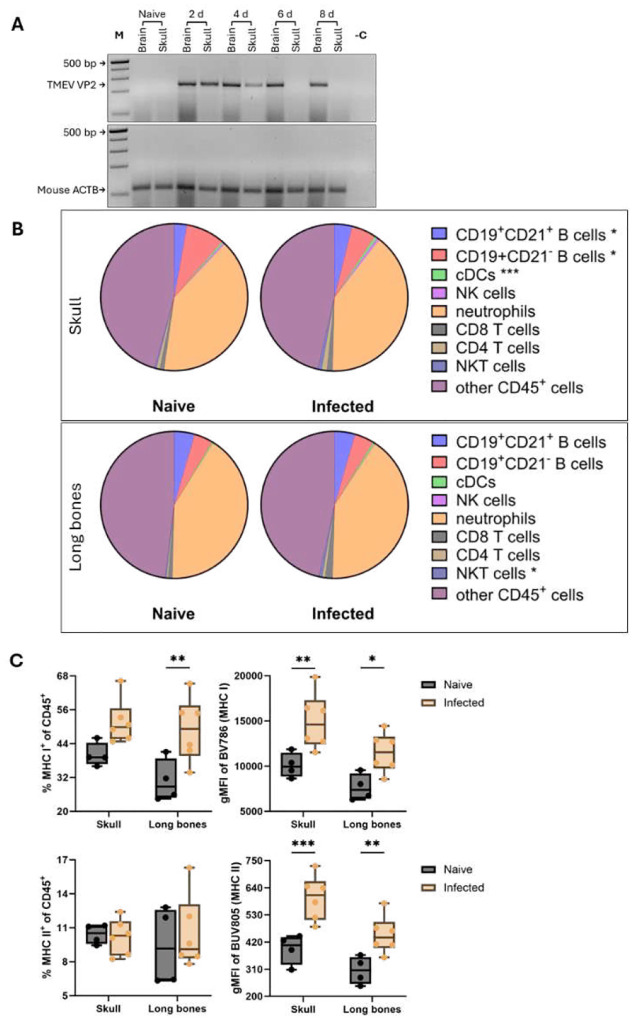
The skull bone marrow harbors virus and is a site for viral antigen-specific CD8 T cells expansion post neurotropic virus infection. (**A**) PCR amplification of TMEV VP2 (top panel) in the brain and skull BM of i.c. inoculated C57BL/6 mice for 2, 4, 6 and 8 days. Brain and skull BM cDNA from an uninfected animal were used as experimental controls. The house-keeping gene β-actin (ACTB) was used as an internal control (bottom panel). -C: template-free negative control, M: marker 100bp DNA ladder. (**B**) Pie charts of CD45+ immune cell composition in skull BM (Top) and long bones marrow (bottom) depicting cellular changes in the hematopoietic compartment following intracerebral TMEV infection in C57BL/6 mice at 7dpi. The mean frequency of each of the listed CD45+ immune cell type was compared between naïve group (n= 4) and TMEV infected group (n= 6) using two-tailed unpaired t-test. (**C**) The box plot shows the difference between naïve and TMEV-infected mice in the frequency of MHC class I^+^ cells and MHC class II^+^ cells of CD45^+^ cells (left) and expression level of MHC class I and class II (right) in skull BM and long bone marrow at 7 dpi. The difference in means was analyzed using two-tailed unpaired t-test, and considered statistically significant if *p* value ≤ 0.05, *: p ≤ 0.05, **: p ≤ 0.01, ***: P ≤ 0.001, ****: P ≤ 0.0001.

Skull BM is a recognized anatomical site for immune cell activation and expansion in response to CNS infection. We therefore analyzed skull BM immune cell populations during acute TMEV infection. First, to determine whether TMEV infection is localized to the brain or disseminates outwards to the skull BM, wild-type C57BL/6 mice were inoculated i.c. with 2×10^6^ pfu of Daniel's strain of TMEV. Brain tissues and skull BM were harvested at 0 (naïve), 2, 4, 6, and 8 days post inoculation (dpi) for RNA isolation and rt-PCR. Although PCR-detectable virus was present in the brain at all timepoints of infection, skull BM harbored virus at 2 and 4 dpi, after which the virus was beyond detectable levels (Fig. 1A). To rule out the potential of introducing the virus into the skull via the i.c. route, we administered TMEV intraperitoneally (i.p.) and screened skull BM, brain, and peripheral BM for the presence of the virus. Again, TMEV was readily detectable, albeit with a weaker signal, in the skull BM at 2 dpi, despite the absence of the virus in the brain and long BM at all timepoints (Fig. S1A and S1B). This indicates that skull BM may be vulnerable to neurotropic viruses regardless of route of entry.

We then examined the impact of intracranial infection on the immune cell composition in the hematopoietic CD45+ compartment of BM in skull and long bones at 7 dpi, using spectral flow cytometry. Few immune cell types were found to be altered in frequency during acute TMEV infection, including B cells and dendritic cells (DCs) in skull BM and NK T cells in peripheral BM (Fig. 1B). Unique to skull BM, the proportion of CD11c+ MHC class II+ DCs significantly increased 1.5-fold after a week of infection. Acute TMEV infection also resulted in increased expression of MHC class I and class II, with an apparent heightened frequency of MHC class I-expressing cells in both skull and peripheral BM (Fig. 1C). These changes reflect how skull BM respond differently to neurotropic viral infection than peripheral BM.

### Virus-specific CD8 T-cells expand in skull BM, brain, and peripheral immune tissues concurrently

**Fig. 2.**
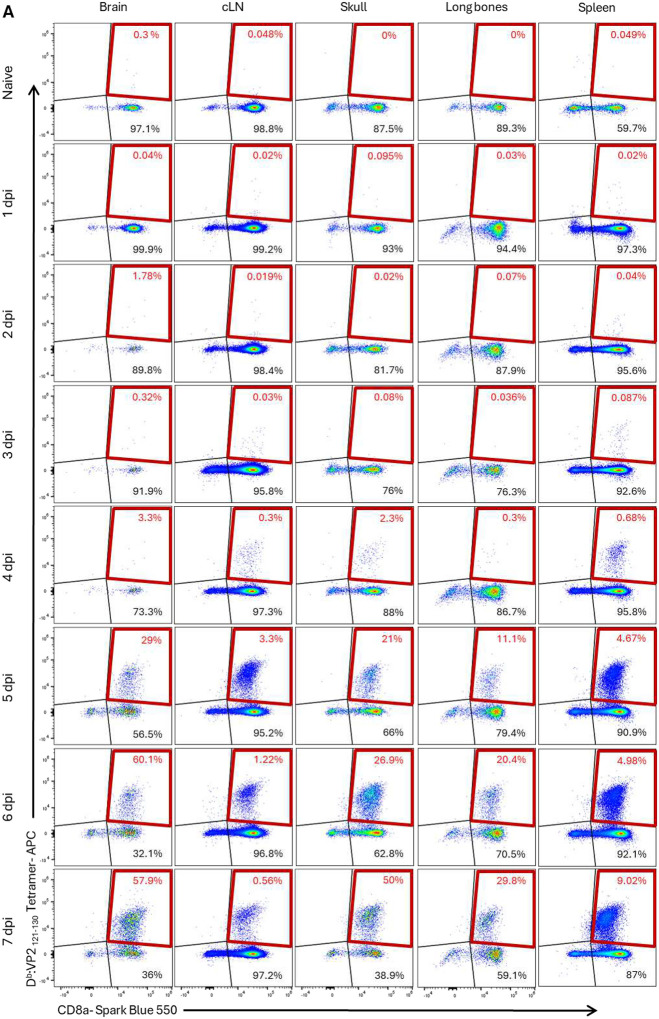

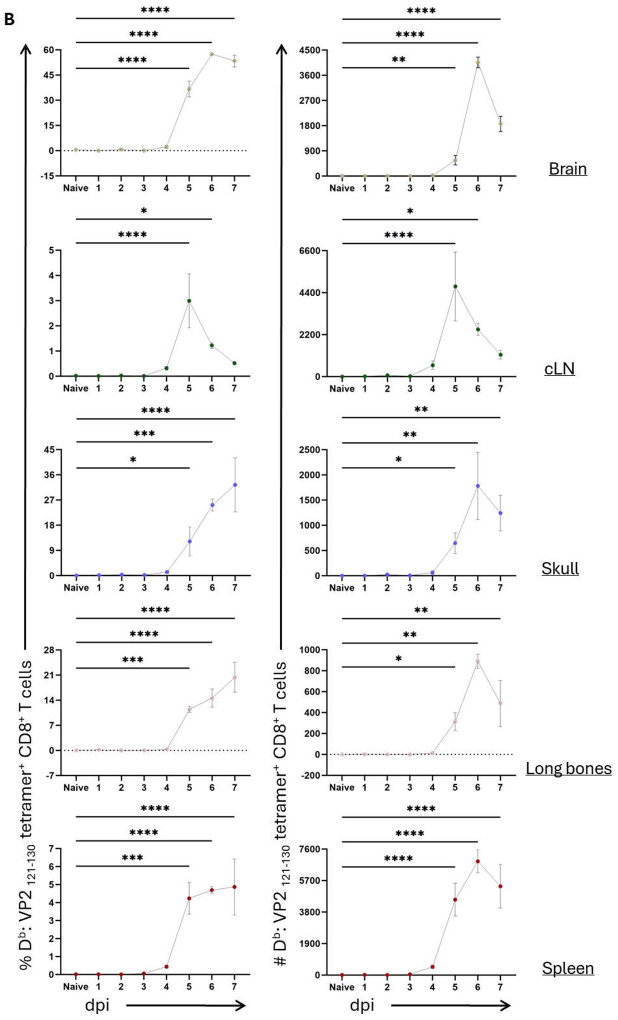
Virus antigen-specific CD8 T cells expand in skull BM at a rate similar to peripheral immune compartments. In a 7-day longitudinal study, wild-type C57BL/6 mice were i.c. inoculated with 2×10^6^ pfu DA TMEV and the brain, cLN, skull BM, long bones marrow and spleen tissues were collected and stained for H-2D^b^: VP2_121–130_ epitope complex tetramer for flow cytometry analysis. (**A**) Representative flow cytometry gates for CD8a and H-2D^b^: TMEV VP2 _121–130_ tetramer on the abovementioned tissues harvested from naïve (uninfected) mice and mice infected for 1, 2, 3, 4, 5, 6 and 7 dpi. Three mice were used for each timepoint. (**B**) Daily frequencies (left) and numbers (right) of virus-specific CD8+ T cells during a week of infection in the tissues analyzed and compared to naïve group using one-way ANOVA with multiple comparison correction. Data is presented as mean ± SEM, and differences to naïve group are considered statistically significant if *P* value ≤ 0.05, *: *P* ≤ 0.05, **: *p* ≤ 0.01, ***: *P* ≤ 0.001, ****: *P* ≤ 0.0001.

To determine whether brain viral infection results in the generation of antiviral CD8 T cells in skull BM we stained for H-2D^b^:VP2_121–130_ tetramer in the infected brain, skull BM, and peripheral immune tissues. Tetramer-positive CD8 T cells were analyzed in TMEV-infected mice for 0 (naïve), 1, 2, 3, 4, 5, 6, and 7 days. Representative gating strategy across multiple anatomical sites is displayed in Figure S2, and staining plots of tetramer-positive CD8 T cell populations during the 7-day infection time course are presented in Figure 2A. We observed a temporal expansion of TMEV-specific CD8 T cells directed against the immunodominant epitope H-2D^b^-restricted VP2_121–130_ in the infected brain, skull BM, and peripheral immune tissues during the initial 7 days of infection (Fig. 2A and 2B). A small yet noticeable population of virus-specific CD8 T cells was detected in all animals as early as 3 dpi in cLN and spleen, and at 4 dpi in skull BM, brain, and long BM. However, the frequency and number of virus-specific CD8 T cells rose significantly at 5 dpi compared to naïve mice and remained significantly elevated at 7 dpi in all tissues (Fig. 2B). Indeed, virus-specific CD8 T cells exhibited a rapid expansion in skull BM in frequency of total CD8 T cells from an average of 1.3% at 4 dpi to 12.2% at 5 dpi, doubling to 25.2% at 6 dpi and 32.4% at 7 dpi, with no numerical difference in total CD8 T cells over time. The frequency of virus-specific CD8 T cells at 6 dpi in skull BM showed the least intragroup variation. Similarly, average number of skull-derived virus-specific CD8 T cells increased by over 10-fold between 4 and 5 dpi and tripled between 5 and 6 dpi. By day 7, virus-specific CD8 T cells constituted about one third of CD8 T cells in skull BM as opposed to over half of brain-infiltrating CD8 T cells. In contrast less than 5% of CD8 T cells in secondary lymphoid tissues were specific to TMEV antigen. Interestingly, peripheral (i.p.) infection with TMEV also resulted in the expansion of virus-specific CD8 T cells in skull BM, brain, and long BM at 7 dpi (Fig. S1C to S1D). Skull BM and brain exhibited higher frequencies of virus-specific CD8 T cells than long BM (Fig. S1E). These findings suggest that neurotropic viral infections can result in the expansion of virus-specific CD8 T cells within skull BM, regardless of route of entry.

**Fig. 3.**
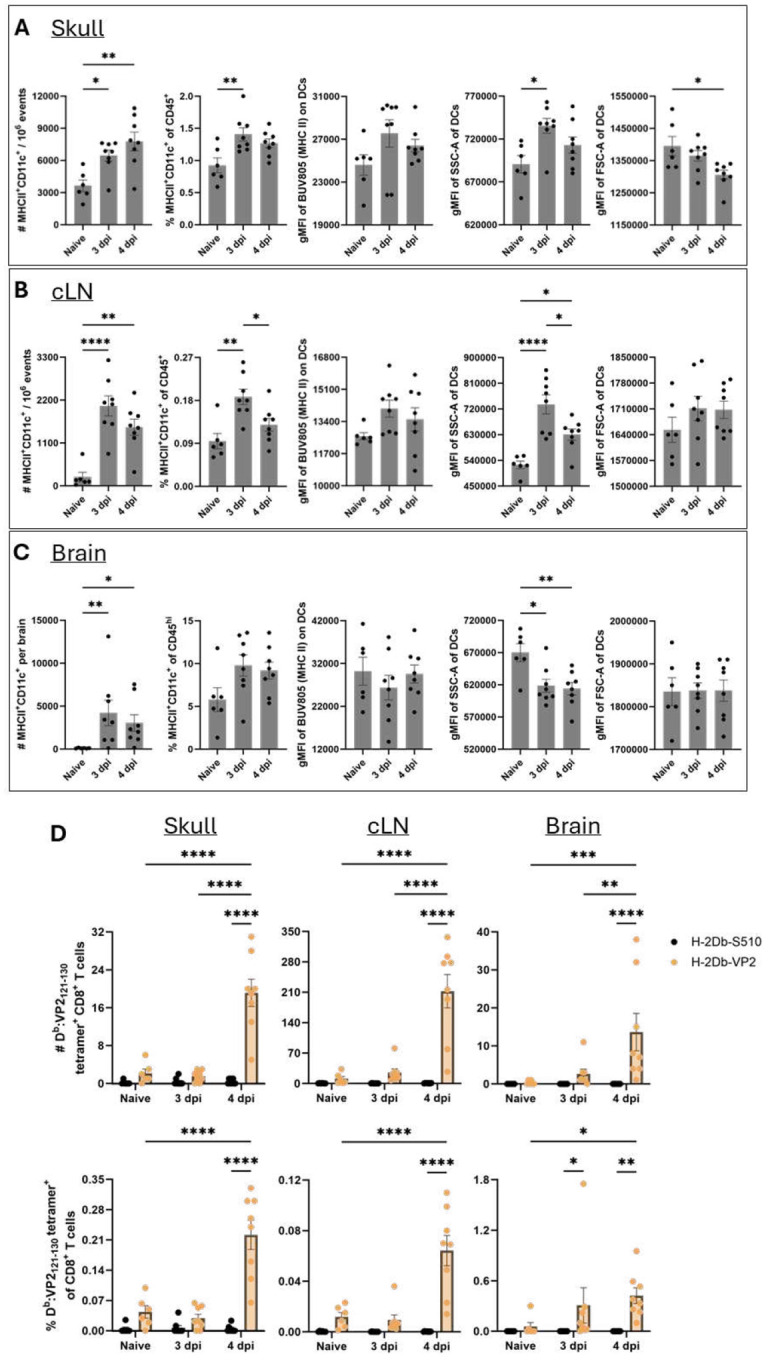
CD11c+ MHC II+ Dendritic cells and virus-specific T cells rapidly expand in skull BM during early neurotropic virus infection. To determine the earliest time point of expansion of virus-specific CD8 T cells, 3 large groups of wild-type C57BL/6 mice (n=6–8/ group, male-to-female ratio= 1:1) were used to stain skull BM, cLN, and brain tissues at 3 and 4 dpi and at naïve state. Numbers, frequencies, and geometric mean of ssc-a and fsc-a of DCs (MHC II+ CD11c+) in naïve mice, and at 3 and 4 dpi in (**A**) skull BM, (**B**) cLN, and (**C**) brain. Comparisons were made using one-way ANOVA analysis with multiple comparison correction. (**D**) Numbers (top) and frequencies (bottom) of virus-specific CD8 T cells (H-2D^b^: VP2_121–130_ tetramer; yellow) at 3 and 4 dpi in skull BM, cLN, and brain. H-2D^b^ complexed with D^b^-restricted irrelevant epitope (MHV S510) tetramer (black) served as specificity control for tetramer staining. Data was analyzed using two-way ANOVA followed by Šídák's multiple comparisons test. Bar graphs show individual values with mean ± SEM. *: *P* ≤ 0.05, **: *p* ≤ 0.01, ***: *P* ≤ 0.001, ****: *P* ≤ 0.0001.

Given our observation of early expansion of virus-specific CD8 T cells at 3 dpi, we conducted a more focused study with a large group of sex- and age-matched animals comparing changes in immune cell composition and virus-specific CD8 T cells in the brain, cLN, and skull BM in mice infected for 3 and 4 days versus naïve animals. First, we evaluated the impact of TMEV infection on CD11c+ MHC II+ DCs (Fig. 3A to 3B). While the expression level of MHC class II (geometric mean fluorescence intensity (gMFI) of BUV805) on CD11c+ MHC II+ DCs remained unchanged, the number of DCs rose in all tissues on day 3. This was accompanied by increased frequency and elevated gMFI of side scatter (ssc) of CD11c+ MHC II+ DCs in skull BM (Fig. 3A) and cLN (Fig. 3B). Conversely, gMFI of ssc of brain DCs was significantly diminished during early TMEV infection (Fig. 3C). The various changes induced by TMEV infection on the DC populations indicate that DCs at different sites display unique physical characteristics following viral infections.

Minimal changes were observed in immune cell composition of the hematopoietic compartment in skull BM during the first 4 days of infection, impacting mainly innate immune cells. Infection-induced changes included numerical alterations favouring the H-2K^b^ expressing cells, with increased frequency and number at 3 and 4 dpi, respectively (Fig. S3A). Other changes impacted TCRβ+ NK cells (NK T cells) (Fig. S3B) and TCRβ- NK cells (Fig. S3C). NK T cells expanded at 3 and 4 dpi, and the number and frequency of granzyme B+ NK T cells dramatically surged at 3 dpi in skull BM (Fig. S3B). Similarly, NK cells exhibited phenotypical changes as the number and frequency of granzyme B+ NK cells and CD69+ NK cells more than doubled at 3 dpi (Fig. S3C).

Next, we aimed to identify the rate by which virus-specific CD8 T cells expand in the skull BM following brain viral infection, and how that compares to their expansion in the infected brain and cLN. Staining for H-2D^b^: VP2_121–130_ tetramer and negative control H-2D^b^: S510 tetramer substantiated our previous observation of notable and consistent expansion of virus-specific CD8 T cells in skull BM, cLN, and infected brain at 4 dpi (Fig. 3D). Paired analysis showed higher proportions of CD8 T cells in skull BM than in cLN directed against TMEV at 4 dpi despite greater absolute counts of virus-specific CD8 T cells in the cLN (Fig. S4A and S4B). There was no sex difference in the number and frequency of virus-specific CD8 T cells at this timepoint.

We then examined the phenotype of skull-derived virus-specific CD8 T cells generated at 4 dpi. Two numerically dominant subsets were identified based on their expression of the activation marker CD69 and granzyme B: the granzyme B+ CD69− subset and the double-negative subset (Fig. S4C), which paralleled the phenotype of virus-specific CD8 T cells in the cLN (Fig. S4E). In contrast, tetramer-negative CD8 T cells in skull BM were primarily double-negative for the two markers (Fig. S4D). However, there was a strong positive correlation (Spearman r= 0.886, *P* value= 0.006) between the number of Granzyme B+ virus-specific CD8 T cells and the number of tetramer-negative CD8 T cells expressing Granzyme B+ in skull BM (Fig. S5A), indicating that the cytotoxic phenotype of virus-specific CD8 T cells may be a marker of a broader activation of antigen-specific and -nonspecific CD8 T cells in skull BM. This correlation was also observed in the brain but not in the cLN, suggesting that skull BM can mirror some of the inflammatory aspects in the CNS. Additionally, virus-specific CD8 T cells exhibited more pronounced expression of granzyme B than NK cells and NK T cells in skull BM at 4 dpi (Fig. S5B). Virus-specific CD8 T cells expressing granzyme B constituted a larger proportion of overall skull BM CD8 T cells than cLN CD8 T cells, however a smaller proportion than brain CD8 T cells (Fig. S5C and S5D). Similarly, virus-specific CD8 T cells expressing CD69 were less frequent in skull BM than in the brain (Fig. S5E). Furthermore, most of skull BM virus-specific CD8 T cells coexpressed CD44 and CD62L (Fig. S4F), resulting in a distinct phenotype that distinguished VP2_121–130_-specific CD8 T cells from other CD8 T cells in skull BM (Fig. S4G). Virus-specific CD8 T cells in skull BM, therefore, varied in surface markers related to effector state at early timepoints of infection.

### Early virus-specific CD8 T cells require MHC class I expression on APCs to expand in skull BM

Considering that skull BM harbored TMEV during the initial 4 days of brain infection (Fig. 1A) and displayed an expanded population of Granzyme B+ virus antigen-specific CD8 T cells (Fig. S5) as early as 4 dpi, we aimed to identify APCs contributing to the priming and activation of TMEV-specific CD8 T cells in skull BM. Our previous studies, using the Cre−lox system for the conditional deletion of single MHC class I molecules, have demonstrated that MHC class I-restricted TMEV antigen presentation on discrete APCs is essential for the priming of brain infiltrating antiviral CD8 T cells^27–30^. Using these established cre lines in pilot experiments, we asked what APCs were involved in the expansion of antiviral CD8 T cells in skull BM. To answer this question, we first looked at the involvement of two relevant types of cells, dendritic cells and endothelial cells. Given that skull-meningeal conduits are CD31+, it is plausible that endothelial cells can serve as an APC subset that activates virus-specific CD8 T cells in skull BM. Indeed, utilizing our previously established *Cdh5-Cre:* H-2D^b^ LoxP mice, we found that virus-specific CD8 T cells in skull and peripheral BM were abolished when H-2D^b^ was conditionally deleted in CDH5+ cells (*Cdh5-Cre*+ mice), compared to *Cdh5 Cre−* mice at 7 dpi (Fig. S6A and S6B). Lack of H-2D^b^ on CDH5+ cells also negatively impacted the expansion of different subsets of virus-specific CD8 T cells in skull BM, namely the CD44+CD62L− subset and the Ly6C+ subset (Fig. S6C), depicting a pivotal role for antigen presentation by CDH5+ cells in the generation of various subsets of skull BM-derived virus-specific CD8 T cells during acute brain viral infection.

**Fig. 4.**
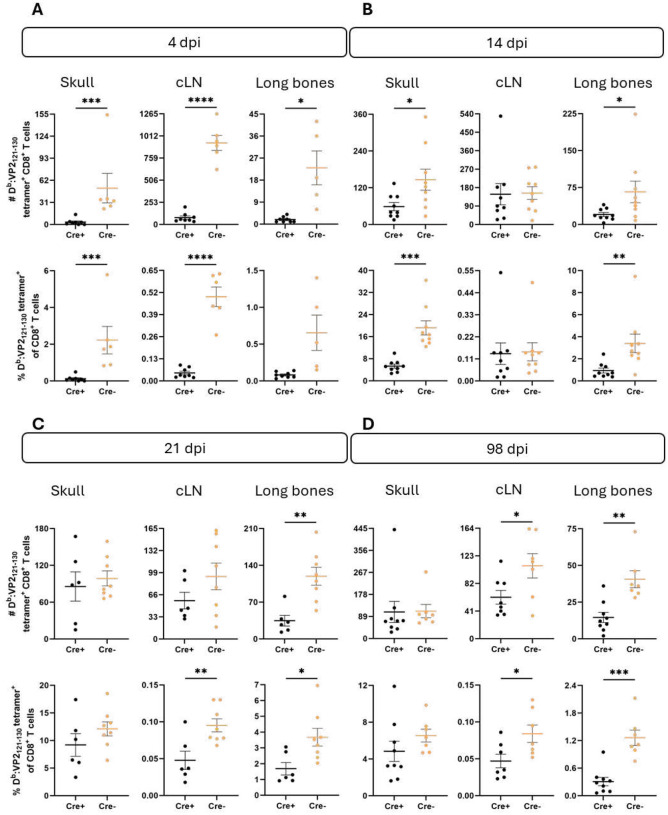
H-2D^b^- restricted antigen presentation on CD11c+ APCs is required to generate virus epitope-specific CD8 T cells during acute and subacute brain viral infection but not to maintain these cells in skull BM during established memory. (**A-D**) The impact of conditional depletion of H-2D^b^ on CD11c-expressing APCs on numbers and frequencies of virus epitope-specific CD8 T cells at (**A**) 4, (**B**) 14, (**C**) 21, and (**D**) 98 dpi, in skull BM, cLN, and peripheral BM. Data was tested for normality and analyzed using two-tailed unpaired t- test or Mann-Whitney test. Individual values are plotted with mean and SEM. *: *P* ≤ 0.05, **: *p* ≤ 0.01, ***: *P* ≤ 0.001, ****: *P* ≤ 0.0001.

Furthermore, CD11c+ MHCII+ DCs were among the few immune cell types exhibiting TMEV infection-induced alterations during acute infection across multiple anatomical sites including skull BM (Fig. 1B and 3). Thus, C57BL/6 mice with conditional deletion of H-2D^b^ on CD11c+ APCs (CD11c-Cre: MHC class I KO × D^b^ loxP)^27^ (Fig. S7A) were infected with TMEV to assess the requirement of H-2D^b^-restricted antigen presentation by CD11c+ APCs for the generation of virus-specific CD8 T cells in skull BM. Virus-specific CD8 T cells were analyzed during acute infection (4 dpi), subacute infection (14 dpi), early chronic phase (21 dpi), and memory phase (98 dpi) in skull BM, cLN, and long BM. The number and frequency of skull BM-derived CD8 T cells recognizing H-2D^b^-restricted TMEV antigen, VP2_121–130_, were significantly abolished at 4 and 14 dpi in CD11c Cre+ mice relative to CD11c Cre− mice (Fig. 4A and 4B), while remaining unchanged at 21 and 98 dpi (Fig. 4C and 4D). This effect was confined to VP2_121–130_-specific CD8 T cells, as no difference in the number and frequency of other (tetramer-negative) CD8 T cells and total CD8 T cells was seen between CD11c Cre+ and CD11c Cre− mice. The effect of the conditional deletion of H-2D^b^ on virus-specific CD8 T cells was most pronounced at 4 dpi, with the average number and frequency ~ 15- and 18-fold lower in CD11c Cre+ mice, respectively (Fig. 4A). VP2_121–130_-specific CD8 T cells in peripheral BM exhibited a significant decrease in number and frequency across all timepoints of the study in CD11c Cre+ mice compared to CD11c Cre− mice (Fig. 4A to 4D), with the largest reduction occurring at 4 dpi (Fig. 4A). Similarly, VP2_121–130_-specific CD8 T cells in cLN were numerically perturbed in CD11c Cre+ mice at all timepoints except at 14 dpi (Fig. 4A to 4D). Paired analysis between different anatomical sites revealed that skull BM contained a higher frequency of virus-specific CD8 T cells than cLN across all timepoints in CD11c Cre− mice, and at 14, 21, and 98 dpi in CD11c Cre+ mice (Fig. S8A). Notably, virus-specific CD8 T cells in skull BM and peripheral BM were more elevated at 98 dpi than during initial expansion at 4 dpi, whereas they were markedly declined in cLN at 98 than at 4 dpi (Fig. S7C). Collectively, these data point to antigen-presentation by H-2D^b^- expressing CD11c+ APCs being integral in the generation of virus-specific CD8 T cells in skull BM during acute and subacute phases of brain infection, and in peripheral lymphoid tissues during acute, subacute, and long after infection clearance.

We next determined the impact of H-2D^b^ deletion in CD11c+ APCs on the phenotype of virus-specific CD8 T cells in skull BM. Skull BM in CD11c Cre+ mice contained significantly lower number and frequency of CD44+CD62L− and CD44+CD62L+ antiviral CD8 T cells at 4 dpi. Over time, CD44+CD62L+ population emerged as the predominant subset of skull-derived virus-specific CD8 T cells and were negatively impacted in CD11c Cre+ mice at all timepoints, except at 21 dpi (Fig. S9A). Based on the co-expression of CD69 and CD103, the CD69-CD103− subset was predominant at 4 and 14 dpi (Fig. S9B). This double negative subset was significantly lower in CD11c Cre+ mice in number at the three earliest timepoints and in frequency across all timepoints. The CD69+CD103− subset was also reduced in CD11c Cre+ mice at 14 dpi. The CD69-CD103+ subset was unique in that it displayed no difference in abundance between CD11c Cre+ and CD11c Cre− mice; however, this subset increased to frequencies surpassing those of CD69+CD103− at 21 dpi. All subsets, except for CD69-CD103+, were markedly diminished at 98 dpi in CD11c Cre+ mice relative to CD11c Cre− mice (Fig. S9B). These observations imply that virus-specific CD8 T cells in skull BM, primed by H-2D^b^ expressing CD11c+ APCs, possess a highly dynamic temporal expression of distinct activation surface markers.

**Fig. 5.**
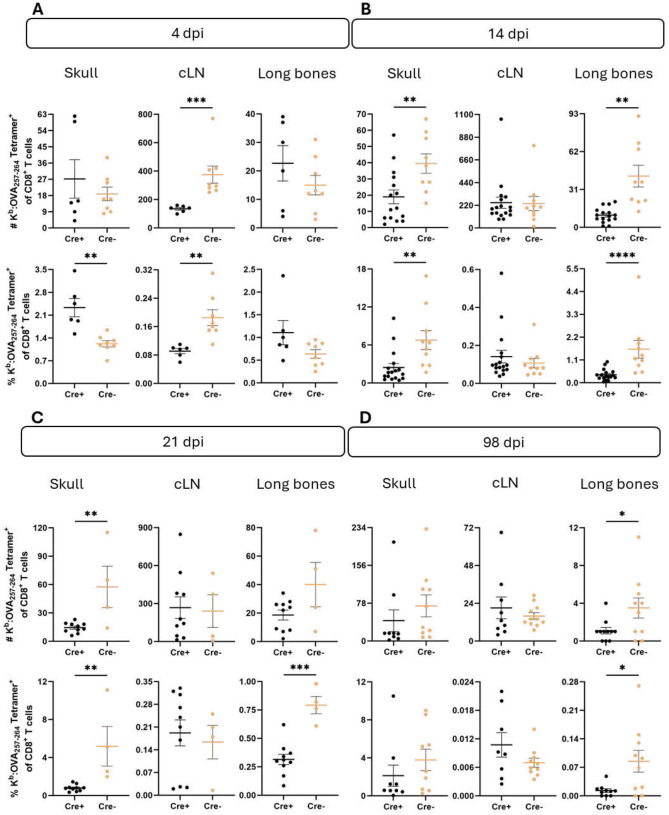
H-2K^b^-restricted antigen presentation on CD11c-expressing APCs is required to generate virus epitope-specific CD8+ T cells during later timepoints of viral infection but not for the persistence of these cells in the skull BM. (**A-D**) The impact of conditional depletion of H-2K^b^ on CD11c+ APCs on numbers and frequencies of virus epitope-specific CD8+ T cells at (**A**) 4, (**B**) 14, (**C**) 21, and (**D**) 98 dpi, in skull BM, cLN, and peripheral BM. Data was tested for normality and analyzed using two-tailed unpaired t- test or Mann-Whitney test. Individual values are plotted with mean and SEM. *: *P* ≤ 0.05, **: *p* ≤ 0.01, ***: *P* ≤ 0.001, ****: *P* ≤ 0.0001.

To determine whether the conditional deletion of the other MHC class I molecule in C57BL/6 mice; H-2K^b^, in CD11c+ APCs also resulted in impaired expansion of antiviral CD8 T cells in skull BM, C57BL/6 mice with conditional deletion of H-2K^b^ on CD11c+ APCs (CD11c-Cre. MHC class I KO × K^b^ loxP)^29^ (Fig. S7B) were infected with ovalbumin-encoding TMEV. CD8 T cells against H-2K^b^-restricted Ova antigen (Ova_257–264_; SIINFEKL) were analyzed in skull BM, cLN, and long BM at 4, 14, 21, and 98 dpi. A slight, yet significant frequency increase of virus-specific CD8 T cells was noted in skull BM of CD11c Cre+ mice compared to CD11c Cre− mice at 4 dpi (Fig. 5A). However, skull-derived virus-specific CD8 T cells were significantly diminished in number and frequency in CD11c Cre+ mice compared to CD11c Cre− mice at 14 and 21 dpi (Fig. 5B and 5C). The most substantial decline occurred at 21 dpi (4-fold decrease in mean number and 7-fold decrease in mean frequency) (Fig. 5C). Conversely, the expansion of cLN-derived virus-specific CD8 T cells was hampered solely during the early phase of acute infection at 4 dpi (Fig. 5A); thereafter, the conditional deletion of H-2K^b^ had no impact on the numbers or proportions of virus-specific CD8 T cells in cLN (Fig. 5B to 5D). Moreover, cLN contained significantly lower proportions of virus-specific CD8 T cells compared to skull BM at all timepoints in both CD11c Cre+ and CD11c Cre− mice (Fig. S8B). In stark contrast to the dynamics observed in cLN, peripheral BM-derived virus-specific CD8 T cells were numerically diminished in CD11c Cre+ compared to CD11c Cre− mice across all timepoints beyond 4 dpi (Fig. 5A to 5D), with the most noticeable decrease of 3-fold in mean number and 7-fold decrease in mean frequency observed at 98 dpi (Fig. 5D). Thus, H-2K^b^-expressing APCs are central for the initial expansion of virus-specific CD8 T cells in cLN during early acute infection, the maintenance of skull-derived virus-specific CD8 T cells during subacute infection and the early phase of chronic infection, and the persistence of long BM-derived virus-specific CD8 T cells throughout acute and chronic infection. This depicts how CD11c+ APCs prime H-2K^b^-restricted antigen-specific CD8 T cells in a spatiotemporal fashion, which is distinct from the priming of H-2D^b^- restricted antigen-specific CD8 T cells. An important observation in CD11c-Cre H-2K^b^ LoxP mice, which fail to eradicate viral infection^31^, was the significantly elevated number of virus-specific CD8 T cells in skull BM at 98 dpi relative to the initial expansion observed at 4 dpi (Fig. S7D). In contrast, peripheral BM and cLN contained significantly less virus-specific CD8 T cells during the chronic phase compared to the acute infection. This suggests that viral persistence in the CNS can be one possible mechanism of skull BM retaining virus-specific CD8 T cells, and that CNS infections uniquely affect skull BM.

H-2K^b^ -expressing CD11c+ APCs were also found to modulate the phenotype of virus-specific CD8 T cells during different phases of the viral infection (Fig. S10). Strikingly, CD44+CD62L− and CD44-CD62L+ subsets of skull-derived virus-specific CD8 T cells were retained at higher frequencies in CD11c Cre+ mice relative to CD11c Cre− mice at 4 dpi (Fig. S10A). The CD44-CD62L+ subset was higher in frequency than the double-negative and double-positive subsets at 4 dpi. The CD44+ CD62L− subset exhibited lower frequency and number in CD11c Cre+ mice at all timepoints except at 4 dpi. This subset also became dominant, particularly in frequency, at later timepoints of infection, namely 21 and 98 dpi (Fig. S10A). Moreover, the double-negative subset based on CD69 and CD103 co-expression was the dominant subtype of skull-derived virus-specific CD8 T cells at 4 and 14 dpi and was numerically diminished in CD11c Cre+ mice at all timepoints beyond 4 dpi (Fig. S10B). All subsets, except for CD69+CD103−, were markedly decreased in CD11c Cre+ mice compared to CD11c Cre− mice at 21 dpi. The CD69+CD103− subset was only impacted during chronic infection at 98 dpi (Fig. S10B). These observations imply that H-2K^b^-restricted antigen presentation play a role in the expansion of discrete subsets of virus-specific CD8 T cells during different timepoints of CNS viral infections.

### Virus-specific CD8 T cells traffic via circulation to skull BM during early acute brain infection

**Fig. 6.**
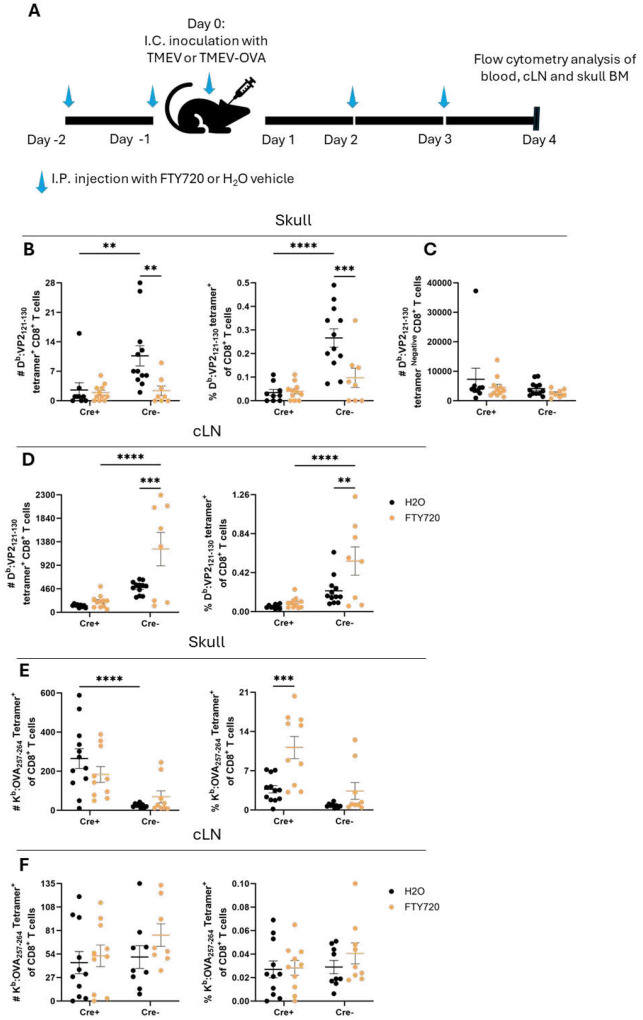
Blocking immune cell trafficking impedes the generation of early wave of H-2D^b^- restricted antigen-specific CD8 T cells in skull BM during acute brain viral infection. (**A**) Experimental setup to determine the extent peripheral immune cells contribute to the generation of early virus-specific CD8 T cells in skull BM. Transgenic mice with conditional depletion of either H-2D^b^ or H-2K^b^ in CD11c+ APCs were i.p. injected twice daily with 100ul of either (0.5mg/kg) FTY720 or water vehicle (control) starting at 2 days prior to intracranial inoculation and continuing till end of the study. Blood, cLN, and skull BM were harvested for flow cytometry analysis at 4 dpi. (**B** and **C**) The impact of FTY720 treatment on the numbers and frequencies of (**B**) virus-specific CD8 T cells activated by H-2D^b^-restricted viral antigen and (**C**) tetramer-negative CD8 T cells in skull BM at 4 dpi. (**D**) The impact of FTY720 on the numbers of cLN-derived virus-specific CD8 T cells activated by H-2D^b^-restricted antigen at 4 dpi. (**E** and **F**) The impact of FTY720 treatment on the numbers and frequencies of virus-specific CD8 T cells activated by H-2K^b^-restricted antigen in (**E**) skull BM and (**F**) cLN at 4 dpi. Data was analyzed using two-way ANOVA followed by Šídák's multiple comparisons test. Graphs show individual values with mean ± SEM. *: *P* ≤ 0.05, **: *p* ≤ 0.01, ***: *P* ≤ 0.001, ****: *P* ≤ 0.0001.

To determine whether virus-specific CD8 T cells activated by CD11c+ APCs were locally primed within skull BM niche or peripherally, we attempted to block the trafficking of immune cells from the peripheral lymphoid organs to the skull BM prior to and during the initial four days of brain viral infection. We administered FTY720 or water (control vehicle) to mice with conditional deletion of H-2D^b^ or H-2K^b^ in CD11c+APCs (Fig. 6A). FTY720 prevents lymphocyte egress from lymph nodes and inhibits their recirculation by interfering with S1P- S1PR signalling^32^. This effect was evident on circulating lymphocytes of both CD11c Cre+ and CD11c Cre− mice, where the number and frequency of B cells, CD4 T cells, and CD8 T cells diminished in the blood of mice administered FTY720, while NK cells remained unaffected (Fig. S11). The number of most of these cell types in skull BM and cLN, however, exhibited no significant difference following treatment with FTY720 (Fig. S12). The proportions of H-2D^b^-negative subset in CD11c+ cells also remained unchanged (Fig. S13A). Of note, FTY720 treatment led to increased numbers of skull BM-derived NK cells in CD11c Cre− mice and a decline in myeloid cells in skull BM of CD11c Cre+ mice (Fig. S11A) indicating that modulation of skull BM immune landscape by FTY720 is affected by CD11c Cre status.

Within skull BM, FTY720 administration led to a considerable decrease in both the number and frequency of virus-specific CD8 T cells primed by H-2D^b^-expressing CD11c+ APCs, reaching values akin to those observed with H-2D^b^ deletion on CD11c+ APCs (Fig. 6B). The impact of FTY720 was restricted to virus-specific CD8 T cells, since the tetramer-negative population remained unaltered following FTY720 treatment in both CD11c Cre+ and CD11c Cre− mice (Fig. 6C). We asked whether FTY720 treatment also altered physical properties of virus-specific CD8 T cells in skull BM. No difference attributed to FTY720 administration was seen in the geometric mean of cell side scatter of virus-specific CD8 T cells in skull BM (Fig. S13C). However, the geometric mean of cell side scatter of various immune cell types in skull BM was significantly enhanced in FTY720 group than in water control group (Fig. S13C). This demonstrates a broad modulatory effect of FTY720 on skull BM immune populations.

In cLN, FTY720 treatment resulted in the accumulation of virus-specific CD8 T cells activated by H-2D^b^-restricted antigen presentation in CD11c+ APCs (CD11c Cre−) (Fig. 6D). There was no effect observed in CD11c Cre+ mice, presumably due to the pre-existing attenuation of virus-specific CD8 T cells brought on by dampened antigen presentation. Notably, these observations were limited to virus-specific CD8 T cells activated by H-2D^b^-restricted viral antigen, as FTY720 treatment showed no effect on the number and frequency of virus-specific CD8 T cells in skull BM activated by H-2K^b^-expressing CD11c+ APCs in CD11c Cre− mice (Fig. 6E). However, the frequency of virus-specific cells in CD11c Cre+ mice was augmented upon FTY720 treatment (Fig. 6E). No effect was seen on the number and frequency of virus-specific CD8 T cells in the cLN in mice with H-2K^b^-expressing or -lacking CD11c+ APCs (Fig. 6F). These observations indicate that virus-specific CD8 T cells activated by H-2D^b^-expressing CD11c+ APCs are rapidly recruited to skull BM following their priming in peripheral lymphoid organs via a trafficking axis that can be blocked by FTY720.

**Fig. 7.**
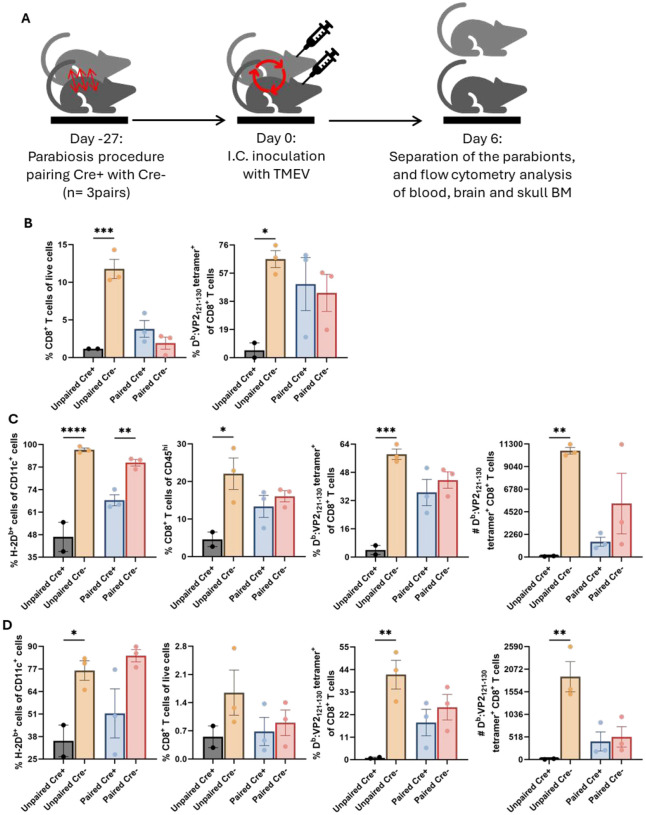
Shared circulation between mice with conditional depletion of MHC class I on DCs and mice with intact expression of MHC class I on DCs rescues production of virus-specific CD8 T cells in skull BM during acute brain viral infection. (**A**) Experimental design for parabiosis study. Three female pairs of parabionts were prepared 4 weeks prior to i.c. inoculation with TMEV. Each pair consisted of a genotypically confirmed CD11c Cre−positive (H-2D^b^ conditionally deleted on CD11c+ cells) joined surgically with a CD11c Cre−negative littermate. Individual CD11c Cre−positive and Cre−negative unpaired mice were used as experimental reference. Blood, brain, and skull BM were collected for flow cytometry analysis at 6 dpi. (**B**) Confirmation of shared circulation in the blood. Frequencies of CD8 T cells and virus-specific CD8 T cells in the blood in CD11c Cre+ and Cre− mice with no prior parabiosis and in CD11c Cre+ and Cre− parabionts after separation. Frequencies of H-2D^b^-expressing cells, CD8 T cells, and numbers of virus-specific CD8 T cells in the (**C**) brain and (**D**) skull BM in the above-mentioned groups. Data was analyzed using two-way ANOVA followed by Šídák's multiple comparisons test. Bar graphs show individual values with mean ± SEM. *: *P* ≤ 0.05, **: *p* ≤ 0.01, ***: *P* ≤ 0.001, ****: *P* ≤ 0.0001.

Since antiviral CD8 T cells activated by H-2D^b^-restricted antigen populate skull BM rapidly after TMEV infection, following their mobilization from outside skull BM, we sought to address the contribution of circulation in the recruitment of virus-specific CD8 T cells to skull BM during early infection. To achieve this, we performed parabiosis in which a mouse with conditional deletion of H-2D^b^ in CD11c+ APCs (Cre+) shared peripheral circulation with a mouse exhibiting intact H-2D^b^ expression in CD11c+ APCs (Cre−) (Fig. 7A). Following 4 weeks of shared circulation, these paired parabionts were infected with TMEV. Individual CD11c Cre+ and CD11c Cre− mice were concurrently infected with TMEV for comparison. All groups were analyzed during acute brain infection at 6 dpi (Fig. 7A). CD11c Cre+ and CD11c Cre− mice showed comparable proportions of circulating CD8 T cells and virus-specific CD8 T cells after sharing circulation (Fig. 7B). Meanwhile, unpaired CD11c Cre+ mice demonstrated a marked decline of these circulating cell types relative to CD11c Cre− mice (Fig. 7B). However, shared circulation did not rescue the proportions of H-2D^b^- expressing cells in the brain of CD11c Cre+ mice (Fig. 7C). Despite that, shared circulation did restore the frequency and number of brain-infiltrating virus-specific CD8 T cells, which were significantly decreased in unpaired CD11c Cre+ mice (Fig. 7C), indicating that priming of brain virus-specific CD8 T cells by H-2D^b^- expressing CD11c+ APCs takes place peripherally during early acute infection. In the skull BM, shared circulation reestablished the frequency of H-2D^b^- expressing cells, and the number and frequency of virus-specific CD8 T cells in paired CD11c Cre+ mice (Fig. 7D). Nevertheless, the number of virus-specific CD8 T cells did not rise to the levels seen in unpaired CD11c Cre− mice.

**Fig. 8.**
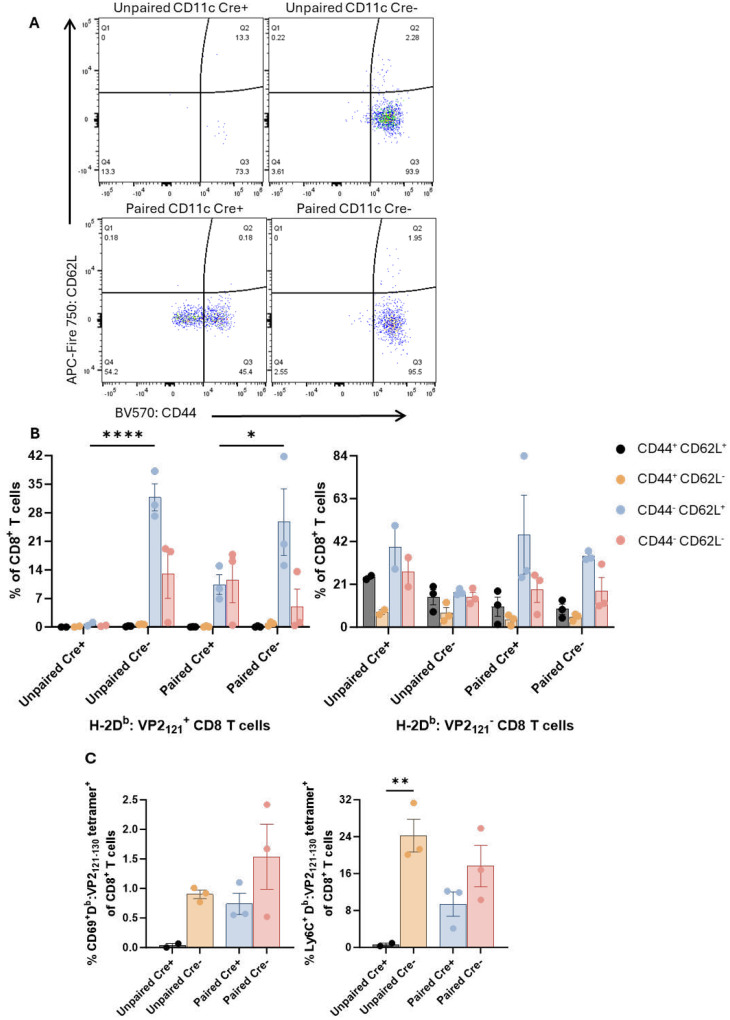
Subsets of virus-specific CD8 T cells are restored in skull BM of CD11c Cre+ after shared circulation with CD11c Cre−. (**A**) Representative flow gates for virus-specific CD8 T cells based on the expression of CD44 and CD62L in individual Cre+ and Cre− mice and parabionts Cre+ and Cre− mice. (**B**) Frequencies of different subsets of virus-specific CD8 T cells and tetramer negative cells in skull BM based on the expression of CD44 and CD62L. Data was analyzed using two-way ANOVA followed by Šídák's multiple comparisons test. (**C**) Frequencies of CD69+ antiviral CD8 T cells and Ly6C+ antiviral CD8 T cells in skull BM in Cre+ and Cre− mice with no prior parabiosis and in parabionts after separation at 6 dpi. Data was analyzed using one-way ANOVA for multiple comparisons. Bar graphs display individual values with mean and SEM bars. *: *P* ≤ 0.05, **: *p* ≤ 0.01, ***: *P* ≤ 0.001, ****: *P* ≤ 0.0001.

Additionally, shared circulation resulted in the recovery of certain subsets of virus-specific CD8 T cells in skull BM (Fig. 8). Interestingly, the frequency of CD44+CD62L− virus-specific CD8 T cells, which was significantly reduced in unpaired CD11c Cre+ mice, did not completely rebound following shared circulation (Fig. 8A and 8B). None of the tetramer-negative subsets exhibited alterations attributable to conditional deletion of H-2D^b^ or to shared circulation (Fig. 8B). However, parabionts showed comparable proportions and numbers of Ly6C+ virus-specific CD8 T cells between CD11c Cre+ and CD11c Cre− mice, unlike diminished proportions in unpaired CD11c Cre+ mice (Fig. 8C). The proportion of CD69+ virus-specific CD8 T cells also appeared to be restored in parabionts despite lacking statistical significance (Fig. 8C). In total, these findings indicate that the generation of antiviral CD8 T cell subsets may be influenced in part by the local environment within skull BM. Moreover, while not reaching statistical significance, unpaired CD11c Cre+ appeared to possess lower number and frequency of CD11c+ MHC II+ DCs in skull BM, and these cells were restored in paired CD11c Cre+ mice (Fig. S14A and S14B). Intriguingly, shared circulation also restored the gMFI of forward-scatter of DCs, which was significantly reduced in unpaired CD11c Cre+ mice (Fig. S14C). Thus, we contend that blood-borne signals are likely necessary to preserve physical properties of DCs in skull BM during early brain infection.

## Discussion

Total BM compartment within various bones, including the flat bones of the skull, exceeds the total volume of all secondary lymphoid tissues, and comprises one of the largest organs^33^. Thus, it is unsurprising that this compartment displays a wide array of functions in health and disease. Skull BM in particular has been increasingly a focus of interest and is now viewed as a major dynamic hub for a diaspora of immune cell populations such as myeloid cells and B lymphocytes^4–6^. Under neuroinflammatory circumstances, highly vascularized bridges between skull BM and brain border pave a trafficking path for immune cells to respond to an injury in the brain^3^. Our chief objective in this study was to define the role of skull BM as a site for the expansion and maintenance of bona fide virus-specific CD8 T cells during brain viral infection using the TMEV model in C57BL/6 mice. We showed here that [1] TMEV infection of the brain renders skull BM susceptible to viral invasion, [2] MHC class I-restricted antigen presentation by CD11c+APCs is required for the initial and early expansion of virus-specific CD8 T cells in skull BM, and [3] virus-specific CD8 T cells activated by H-2D^b^ expressing CD11c+ APCs originate outside skull BM and traffic rapidly via circulation during acute infection to seed skull BM where it persists.

We demonstrated how skull BM displays a distinct response to acute TMEV infection which resulted in an altered DC populations across different anatomical locations, including the brain, skull BM, and cLN, potentially resulting in heterogenous virus-host interactions influenced by the site of viral exposure. Indeed, TMEV was readily detectable in skull BM during early days of infection, regardless of route of entry. This suggests that skull BM is in continuous connection with both the brain and peripheral environment, making it vulnerable to external cues and pathogens. Other recent reports have also highlighted the accessibility of skull BM to viruses or structural components of viruses. Neonate mice i.c. infected with LCMV were found to harbor the virus in skull BM, meninges, and brain tissues as early as 2 dpi^34^, supporting the notion that neurotropic viruses may hijack skull-meninges channels to disseminate to skull BM. In humans, SARS-CoV-2 spike and nucleocapsid proteins were found deposited in postmortem skull BM and skull-meninges channels in individuals who died with acute COVID-19 disease^35^. The co-expression of the viral spike protein and Iba1 in the skull implicates myeloid cells as potential transporters for the virus and virus components between the brain and skull BM. Interestingly, the viral protein persisted in skull BM in some individuals who recovered from the viral infection and showed no PCR detectable virus^35^. Thus, a key imperative is to understand whether the virus in the skull BM reflects active infection of BM cells or blood-borne APCs loaded with viral antigen contributing to the priming of T cells in skull BM. This also raises questions regarding cells that viruses may exploit as trojan horses to invade skull BM.

The rapid spread of the virus to skull BM was accompanied by early expansion of virus-specific CD8 T cells at comparable rates to cLN. We found that the signature phenotype of the majority of early generated antiviral CD8 T cells in skull BM was consistent with T central memory (T_CM_: CD44+CD62L+)^36^ and cytotoxic T cells manifested by granzyme B expression in absence of CD69. This phenotype distinguished VP2-specific CD8 T cells from the remaining CD8 T cells in skull BM, although the extent of cytotoxicity of virus-specific CD8 T cells in skull BM in conferring protection against brain viral infection remains to be defined. The presence of virus-specific CD8 T cells in the BM following viral infection has been shown in humans and mice, particularly in long bones^23–26^. Peripheral BM was proposed to serve as a priming site for T cells during viral infection or exposure to blood-borne antigens, when secondary lymphoid organs are lacking^37,38^. Human peripheral BM has been reported to accumulate CD8 T cells specific for HLA-A*0201–, HLA-B*0801–, HLA-B*0702–, and HLA-B*3501– restricted epitopes derived from common persistent viruses including Epstein-Barr virus and cytomegalovirus (CMV)^23^. CMV-specific CD8 T cells in the BM have been shown to exhibit T_CM_ phenotype and to be distinctive from the phenotype of their counterparts in the blood^39^. In mice, acute LCMV infection results in peak frequencies of virus-specific CD8 T cells (up to 0.5% on average) of BM CD8 T cells at 8 dpi. Not only did these antiviral cells persist for up to a year after acute systemic infection, but they were also functionally protective against LCMV infection upon their transfer into immunocompromised mice^25^.

DCs can either acquire viral antigens from infected cells in the brain and cross-present MHC class I-restricted peptides to CD8 T cells in skull BM and peripheral lymphoid organs, become infected and directly present viral peptides to virus-specific CD8 T cells, or engage in a combination of direct and cross-presentation locally and distally^40–43^. Studies of two-photon intravital microscopy of mouse skull BM illustrated that central memory CD8 T cells interact with DCs within skull BM parenchyma in both an antigen-specific and a nonspecific manner, with the antigen-specific interaction leading to an altered shape and reduced motility of involved T cells^11^. The evidence presented in this paper using conditional transgenic mice underscores the importance of antigen presentation by H-2D^b^ expressing CD11c+ APCs for the expansion of virus-specific CD8 T cells in skull BM during acute and subacute infection.

On one hand, antigen presentation by CD11c+ APCs was not required for the long-term persistence of virus-specific CD8 T cells during established memory phase or chronic infection. On the other hand, antigen presentation by CD11c+ APCs influenced the expression of distinct surface markers typically associated with tissues residency, memory and effector states, most notably the CD44+CD62L− and CD69+CD103+ subsets. The antigen presentation-independent antiviral CD8 T cells in the memory phase may represent a population of antigen-experienced CD8 T cells after having differentiated into memory T cells in skull BM^44^. We also demonstrated that autonomous virus-specific CD8 T cells were unique to skull BM, as virus-specific CD8 T cells in peripheral BM relied on antigen presentation by CD11c+ APCs during memory phase and chronic infection. The mechanisms by which these cells persist in the skull BM remain an open question. During chronic LCMV infection, virus-specific CD8 T cells were found to persist in the BM of long bones due to extensive cell proliferation accompanied by prolonged presence of viral peptides, yet independent of survival signaling mediated by IL-7 and IL-15^45^. Therefore, this maintenance can reflect the host’s attempt to clear a lingering infection and inflammation and maintain established reservoir of long-lived memory cells for robust reactivation during secondary viral exposure, or selective virus dislodging from other tissues. Indeed, antigen-specific T cells stored in peripheral BM were found to reactivate efficiently with no contribution from trafficking cells, as evident by FTY720-mediated blockade of cell migration^46^. Consistent with our observations, Geerman and coauthors observed an abundance of virus-specific CD8 T cells (MHC I-restricted GP_33–41_ and NP_396–404_) in BM tissues, surpassing their frequencies in the spleen during the memory phase long after the resolution of acute LCMV infection^26^. The majority of anti-LCMV CD8 T cells in the BM were CD44+CD62L− but largely negative for CD69 and Ki67, suggesting a preponderance of non-proliferating effector memory CD8 T cells during established memory after viral infection^26^. Additionally, imaging cellular dynamics showed preferential accumulation of central memory CD8 T cells within mouse skull BM at higher proportions than naïve or effector CD8 T cells^47^.

We also noted a peculiar shift in the frequency of virus-specific CD8 T cells between early acute and subacute infection in skull BM of mice with H-2K^b^ deletion on CD11c+ APCs. This could potentially imply a multifaceted dynamic in which antigen presentation by CD11c+ APCs is dispensable during early infection, and other unidentified H-2K^b+^ CD11c-negative APCs take over the activation of virus-specific CD8 T cells with atypical phenotype resulting in their retention within skull BM as a compensatory response. Alternatively, acute TMEV infection in these transgenic mice may induce pronounced inflammation and augmented levels of interferon- α and - β which result in CD69-mediated blockade of sphingosin-1-phosphate receptor 1 (S1P1)^48^ ultimately briefly sequestering antigen-specific CD8 T cells. In support of this view, we observed that antiviral T cells were uniquely augmented in CD11c Cre+ mice with conditional deletion of H-2K^b^ and global deletion of H-2D^b^ upon treatment with FTY720, potentially reflecting an aberrant expression of S1P1 associated specifically with these transgenic mice.

This study answered a critical question about the origin of early waves of virus-specific CD8 T cells in skull BM during acute viral infection of the brain. Pharmacological inhibition of cell egress by FTY720 revealed that virus-specific CD8 T cells activated by H-2D^b^-restricted antigen on DCs could be retained in the cLN and diminished in skull BM. This effect was only seen on virus-specific CD8 T cells as the remaining T cells were insensitive to FTY720 effect. Additionally, FTY720-induced loss of virus-specific CD8 T cells was accompanied by increased granularity of the remaining pool of CD8 T cells, CD4 T cells, and B cells in skull BM, implying that skull BM likely possesses compensatory machinery involving other adaptive immune cells to combat infection. These findings strongly argue that virus-specific CD8 T cells directed against H-2D^b^-restricted antigen in skull BM are primed in peripheral lymphoid organs such as cLN, before mobilizing via S1P- S1PR axis to skull BM during early viral infection of the brain. Importantly, this depicts immune aspects of skull BM that are strikingly different from peripheral BM. In this respect, in newly diagnosed untreated glioblastoma patients, the immune repertoire in skull BM close to the tumor site was found to contain distinctive tumor-reactive CD8 effector T cells that exhibited increased level of S1PR1 expression^49^, potentially rendering them sensitive to FTY720. However, because S1P levels in peripheral BM are lower than plasma levels, creating a gradient that promotes T cell egress from peripheral BM, naïve and antigen-specific T lymphocytes can be retained in the marrow upon the use of FTY720^50^. Because our data offers a brief glance into a highly dynamic milieu, and assuming skull BM contains heterogenous populations of virus-specific CD8 T cells, questions about other possible mechanisms involved in seeding skull BM remain open.

The data from FTY720 study were further corroborated by the results obtained from the parabiosis experiment. We demonstrated that early generated virus-specific CD8 T cells in skull BM are in close contact with blood-circulating virus-specific CD8 T cells, while the unaltered tetramer-negative CD8 T cells are probably sessile. Thus, peripheral circulation facilitates early seeding of skull BM by virus-specific CD8 T cells from other lymphoid organs.

A common theme emerging from these experiments is that the early antiviral CD8 T cells originate outside the skull, with discrete subsets of virus-specific T cells restored upon shared circulation. Admittedly, virus-specific CD8 T cells with central memory phenotype (CD44+ CD62L-) failed to fully reconstitute upon shared circulation. This antiviral subset was also observed to be dependent on antigen-presentation by (CDH5+) endothelial cells. Therefore, CD44+CD62L− virus-specific CD8 T cells may at least partly be generated intrinsically. Thus, it is conceivable that skull BM harbors spatially and potentially functionally diverse populations of virus-specific CD8 T cells, some of which may inhabit niches that are less accessible to peripheral circulation. Indeed, memory CD8 T cells have been shown to extravasate through the microvasculature into mouse skull BM parenchyma, and this movement was mediated by CXCL12 signaling^47^. Whether recruitment via circulation contributes to the replenishment of TMEV-specific memory CD8 T cells in skull BM after infection resolution remains to be explored.

Altogether, this study establishes a role for skull BM in generating and retaining virus-specific CD8 T cells following brain viral infection, reinforcing the dynamic and responsive nature of skull BM to the CNS. The implications of this work could extend to localized manipulation of skull BM environment to further define determinants and fate of the enduring antigen-specific response under common CNS infections. These observations imply that skull BM may be an exciting candidate for vaccines against viral infections of the CNS.

## Materials and methods

### Mice

Wildtype C57BL/6 (Catalogue #: 000664) were purchased from Jackson Laboratory (Bar Harbor, ME). Mice with conditional deletion of H-2D^b^ or H-2K^b^ in CD11c+ cells (*CD11c− Cre* MHC class I^−/−^ mice crossed with H-2D^b^ LoxP or H-2K^b^ LoxP mice) and mice with conditional deletion of H-2D^b^ in CDH5+ cells (*Cdh5− Cre* MHC class I^−/−^ mice crossed with H-2D^b^ LoxP mice) were generated and validated by our group as previously detailed^31,51,52^. These transgenic mice were bred and housed in a barrier room at the Mayo Clinic Animal Research Facility. All animal procedures in this study were conducted in compliance with the protocols approved by Institutional Animal Care and Use Committee of the Mayo Clinic.

### Virus infection

Age and sex-matched mice (8–15-week-old) were anesthetized with isoflurane then intracerebrally (i.c.) inoculated with 2×10^5^ pfu of wildtype TMEV (Daniel’s strain) in serum-free DMEM in total volume of 10 μl. To study CD8 T cell response against H-2K^b^-restricted viral antigen, mice were inoculated with 2×10^4^ pfu of OVA-encoding TMEV (TMEV-OVA). For peripheral infection, mice received intraperitoneally (i.p.) 2×10^6^ pfu of wildtype TMEV in a final volume of 100 μl. Age- and sex-matched control mice were given 100 μl virus-free DMEM. TMEV and TMEV-OVA stocks at the Mayo Clinic were prepared according to previously published protocols^53,54^.

### Staining single cell suspension for flow cytometry

Mice were euthanized at the designated timepoints in each study. One hundred microliter blood was collected in heparinized tubes. Following transcardiac perfusion with PBS, brain, cervical lymph nodes, skull, femurs, tibiae, and spleen were collected in RPMI-containing tubes. Brain, lymph nodes and spleen were subjected to manual homogenization as outlined in prior publications^55,56^. Bones were thoroughly cleaned and each skull was cut into small fragments and placed in a 0.5 mL tube punctured at the bottom. In a similar manner, long bones were cut open at the epiphysis and placed in punctured 0.5 mL tubes. Each tube was placed in a 1.5 mL collection tube and centrifuged to pellet down bone marrow cells according to published protocols^57,58^. Single cell suspensions were passed through a 70-μm cell strainer, followed by lysis of red blood cells using ACK buffer (155 mM ammonium chloride, 10 mM potassium bicarbonate, 0.125 mM EDTA, pH 7.4).

Single cell suspensions were resuspended in PBS and stained for APC- or PE-conjugated H-2D^b^-VP2_121_, H-2D^b^-S510 or H-2K^b^-OVA_257_ tetramers for 30 min at ambient temperature, diluted at 1:100. All tetramers were kindly supplied by the NIH Tetramer Core at Emory University (Atlanta, GA, USA). To exclude dead cells from analysis, cells were stained with Zombie UV (Cat# 423108, Biolegend) for 20 min at room temperature, diluted at 1:1000. Next, cells were incubated with a master mix solution containing antibodies against surface markers and Fc-receptor block CD16/32 (Cat# 553141, BD) for 30 min at 4 C. Cells were then fixed and permeabilized for intracellular staining. A list of the antibodies used in this study is provided in supplemental table 1. Fixed samples were acquired using spectral flow cytometer Cytek Aurora (Cytek, Fremont, CA) to a total of 1×10^6^ events. Data was analyzed using FlowJo 10.8.1 (FlowJo LLC, Ashland, OR).

### RNA isolation and rt-PCR

Total RNA was purified from BM and brain homogenates using TRIzol (Invitrogen, CA, USA)-based extraction, quantified and treated with RNase-Free DNase (RQ1, Cat#: M6101, Promega, WI, USA). One microgram RNA was reverse transcribed using AMV reverse transcriptase-based system (Cat#: A3500, Promega, WI, USA). PCR amplification of the house-keeping gene β-Actin and TMEV VP2 fragment using previously published primers^53^ were performed in a thermocycler using the following conditions: 50°C for 2 minutes, 95°C for 10 minutes followed by 40 cycles of denaturation at 95°C for 15 seconds then annealing at 55°C for 1 minute. Amplicons were run in a 2% agarose gel stained with ethidium bromide.

### Inhibition of peripheral trafficking using FTY720

Mice randomly assigned to the FTY720 treatment group were injected i.p. with 100 μL FTY720 (Cat# SML0700–25mg, Sigma, MA, USA) dissolved in water at 0.15 mg/mL (corresponding to 0.5 mg/kg for an average 30g mouse)^59^. Control mice were injected i.p. with water. All mice received 2 injections of the assigned treatment daily, starting 2 days prior to virus inoculation and continuing until the conclusion of the study and tissue collection.

### Parabiosis

In this study, only female mice were used, due to their inherent lack of aggression and thus favorable survival. After genotyping to confirm the Cre status, female mice with similar weight and age shared a cage for a minimum of 2 weeks before the surgery. Mice pairs were deeply anesthetized using Ketamine/Xylazine (100 and 10 mg/kg, respectively). Mice were given 5mg/kg carprofen and 1mg/kg buprenorphine-SR subcutaneously (s.c.), for analgesia. Mice in the supinated orientation were shaved on the sides adjacent to each other, were incised and joined from elbows to knees as detailed in a previously published methodology paper^60^. Post-surgery, mice were kept in a clean cage on a heating pad, injected s.c. with 0.9% NaCl to maintain hydration and monitored until they woke up and started moving. For the next 5 days, mice had access to water supplied with Baytril to minimize the risk of infection. All pairs were monitored daily. Four weeks after parabiosis, each mouse of the pairs was inoculated i.c. with TMEV. On day 6 post inoculation, mice were euthanized and separated, and tissues were collected for flow cytometry analysis.

### Statistical analysis

Graph Pad Prism software version 10.0.0 was used for statistical tests and graphing data. Statistical tests used for each study are described in figure legends.

## Supplementary Material

1

## Data Availability

All data are available in the main text or the supplementary materials.”
