# Supplementary material for: Virus-specific CD8 T cells rapidly populate and persist in skull bone marrow after brain infection
Source: Res Sq. 2026 Jun 3:rs.3.rs-9890268. Preprint. [Version 1] doi: 10.21203/rs.3.rs-9890268/v1 (PMC13252567; doi:10.21203/rs.3.rs-9890268/v1)
Supplement: 1 [file NIHPPRS9890268V1-supplement-1.pdf]

Supplemental Material

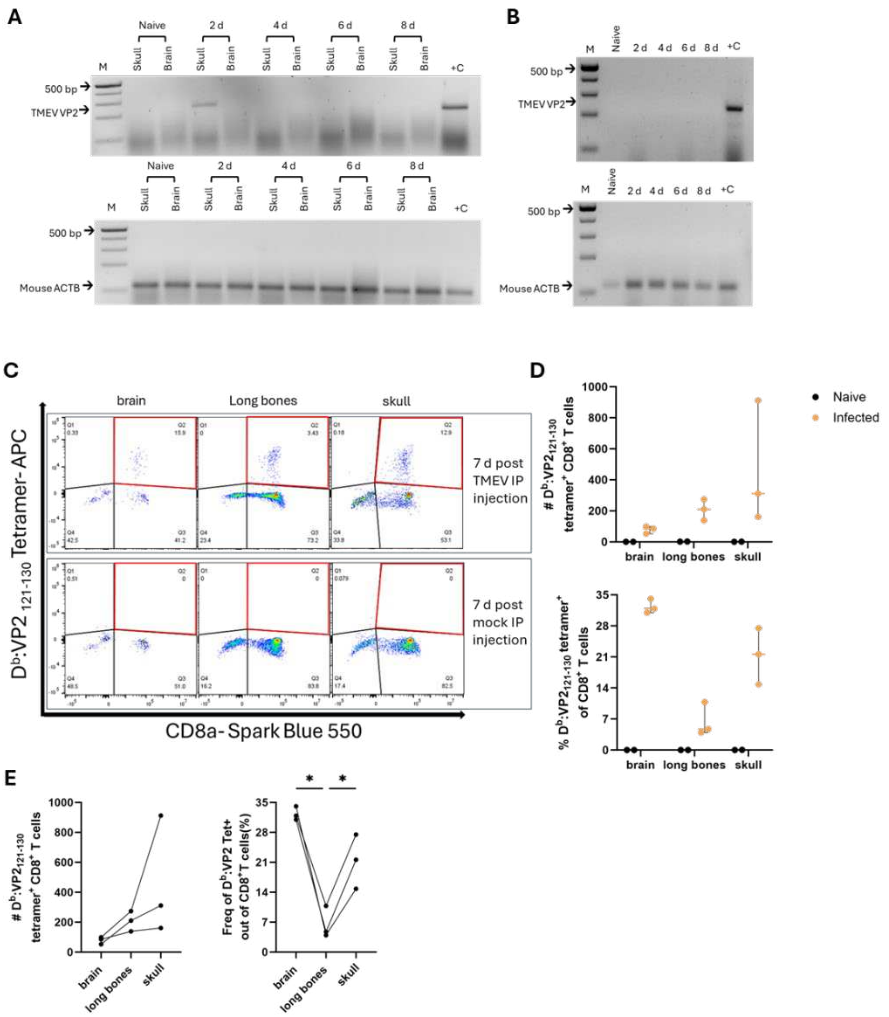

Fig. S1: Skull BM harbors TMEV and TMEV-specific CD8 T cells following peripheral infection. (A) Skull BM, brain, and (B) long bone marrow were screened for TMEV by PCR at 2, 4, 6 and 8 dpi following intraperitoneal inoculation with TMEV. A naïve mouse injected with DMEM was used as a negative control. Mouse ACTB was used as an internal control. M: marker 100bp DNA ladder. +C: brain cDNA from an animal infected with TMEV by i.c. route served as a positive control for VP2 and ACTB. (C) Representative flow cytometry gates for CD8a and H-2D^b: VP2 $_{121\text{-}130}$ tetramer on T cells from the brain, peripheral BM, and skull BM of naïve and infected mice using peripheral route at 7 dpi. TMEV epitope-specific CD8 T cells are demarcated in red-highlighted gates. (D) Unpaired comparison of the frequency and number of virus-specific CD8 T cells between naïve (n=2) and infected mice (n=3) was performed on each organ using Man-Whitney test. Individual values are plotted with bars showing median and range. (E) Frequency and number of virus-specific CD8 T cells at 7dpi of peripherally infected mice were compared between paired brains, peripheral BM and skull BM using repeated measures one-way ANOVA followed by post-hoc Bonferroni.

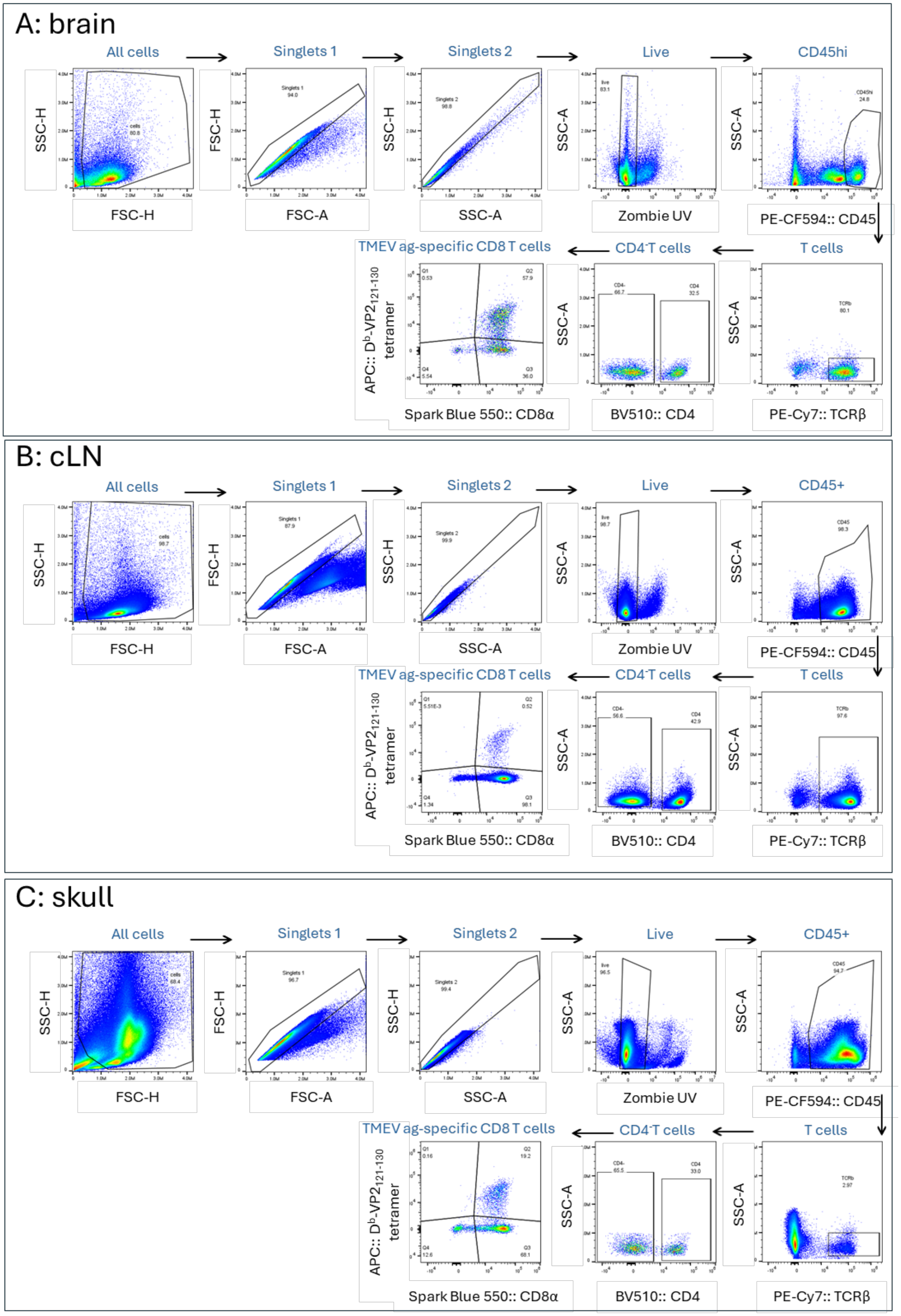

A: brain
All cells
Singlets 1
Singlets 2
Live
CD45hi
SSC-H
FSC-H
FSC-A
SSC-A
Zombie UV
PE-CF594:: CD45
TMEV ag-specific CD8 T cells
CD4^{-}T cells
T cells
APC:: D^{b}-VP2$_{121-130}$ tetramer
Spark Blue 550:: CD8α
BV510:: CD4
PE-Cy7:: TCRβ
B: cLN
All cells
Singlets 1
Singlets 2
Live
CD45+
SSC-H
FSC-H
FSC-A
SSC-A
Zombie UV
PE-CF594:: CD45
TMEV ag-specific CD8 T cells
CD4^{-}T cells
T cells
APC:: D^{b}-VP2$_{121-130}$ tetramer
Spark Blue 550:: CD8α
BV510:: CD4
PE-Cy7:: TCRβ
C: skull
All cells
Singlets 1
Singlets 2
Live
CD45+
SSC-H
FSC-H
FSC-A
SSC-A
Zombie UV
PE-CF594:: CD45
TMEV ag-specific CD8 T cells
CD4^{-}T cells
T cells
APC:: D^{b}-VP2$_{121-130}$ tetramer
Spark Blue 550:: CD8α
BV510:: CD4
PE-Cy7:: TCRβ

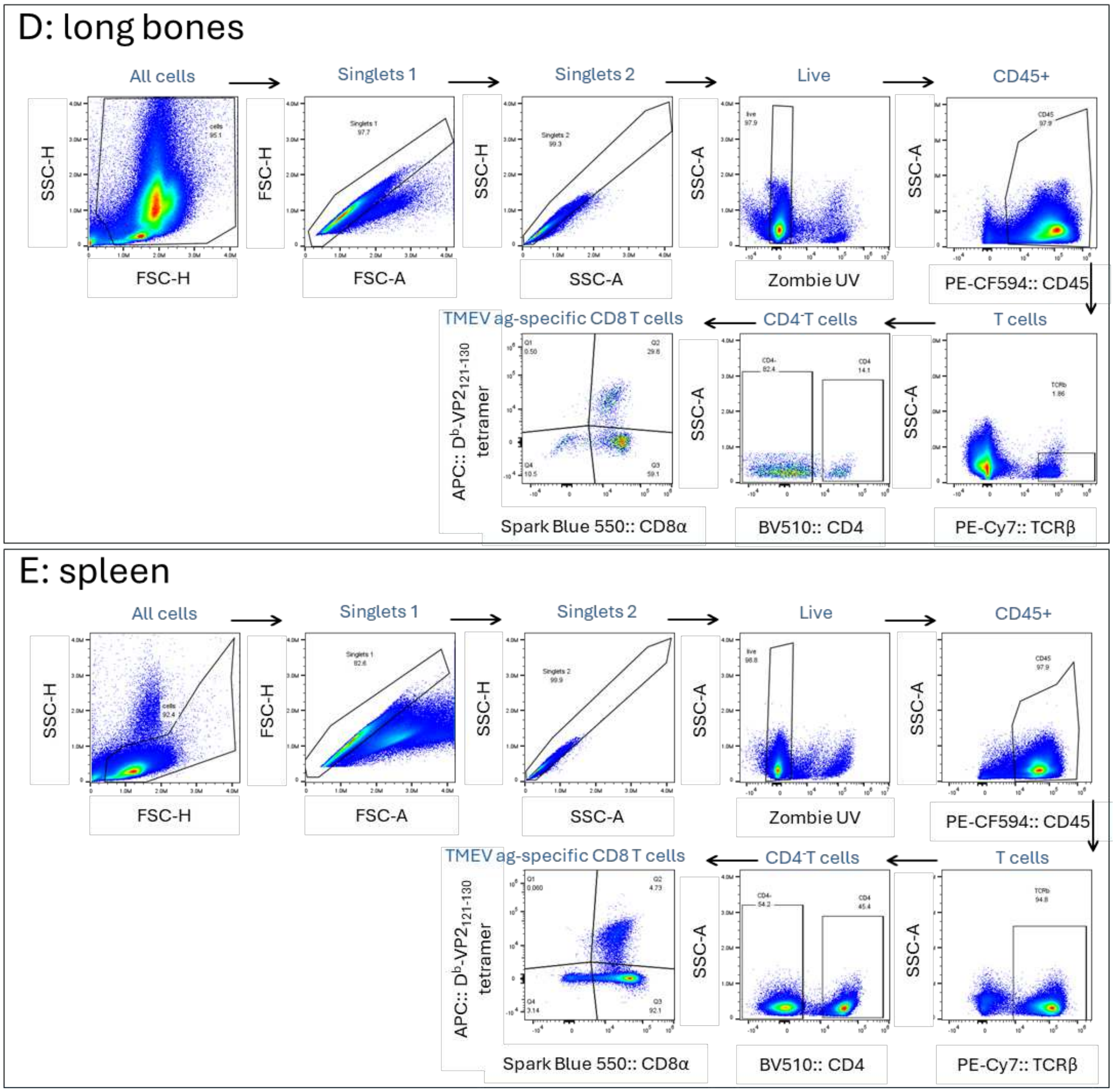

Fig. S2: Virus-specific CD8 T cells expand in skull BM and peripheral lymphoid organs at comparable rates. Gating strategy of TMEV D^b-restricted $VP2_{121\text{-}130}$ epitope- specific CD8 T cells in the (A) brain, (B) cLN, (C) skull, (D) long bones, and (E) spleen.

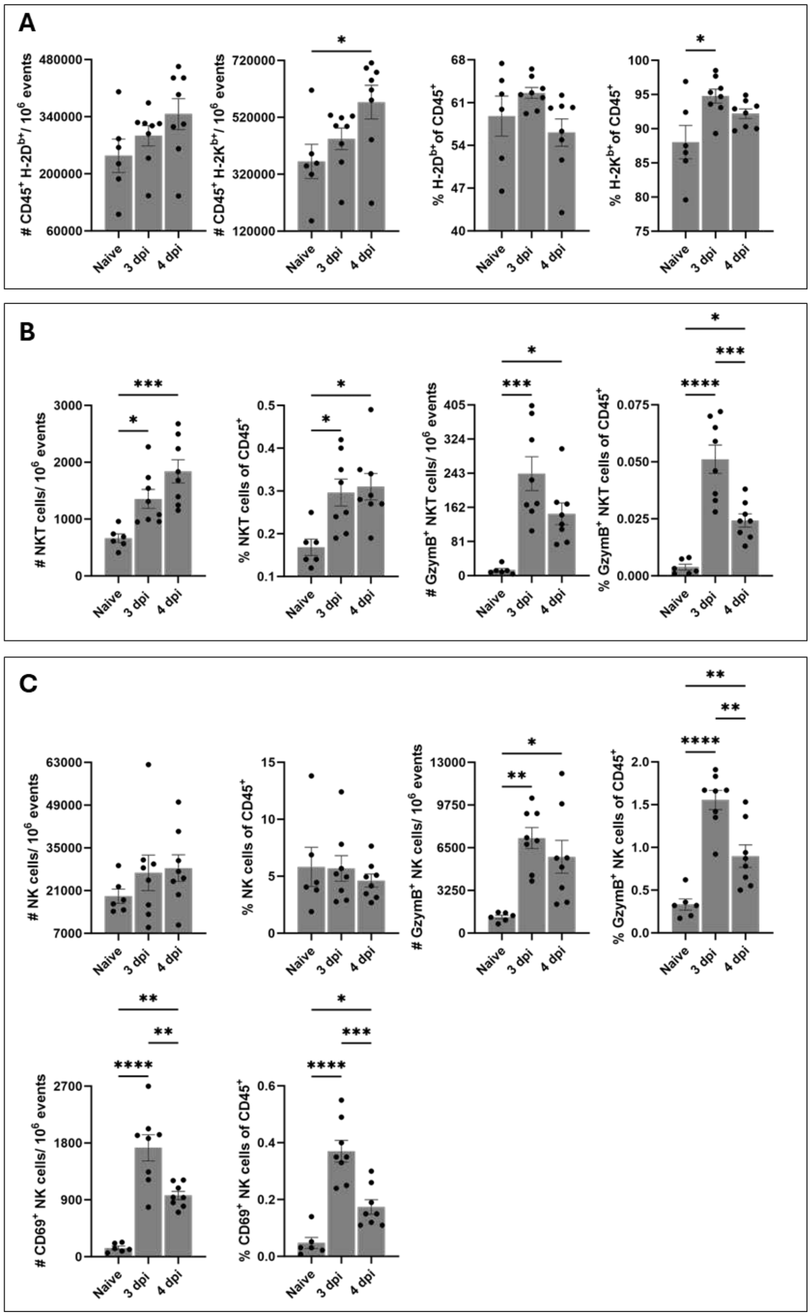

Fig. S3: TMEV infection induces numerical changes in immune cells in skull BM during early timepoints of acute TMEV infection. (A) Number and frequency of H-2D^{b+} cells and H-2K^{b+} cells at naïve state and at 3 and 4 dpi. Number and frequency of (B) NK T cells and Granzyme B^{+} NK T cells, and (C) NK cells, Granzyme B^{+} NK cells, and CD69^{+} NK cells at naïve state and at 3 and 4 dpi. Bar graphs show values from individual mice with mean ± SEM. Data was tested for normality and comparisons were made using ordinary one-way ANOVA with Šídák's multiple comparisons test or Kruskal-Walis with Dunn’s multiple comparison test. Differences were deemed significant if P value ≤ 0.05, *: $P \leq 0.05$, **: $P \leq 0.01$, ***: $P \leq 0.001$, ****: $P \leq 0.0001$.

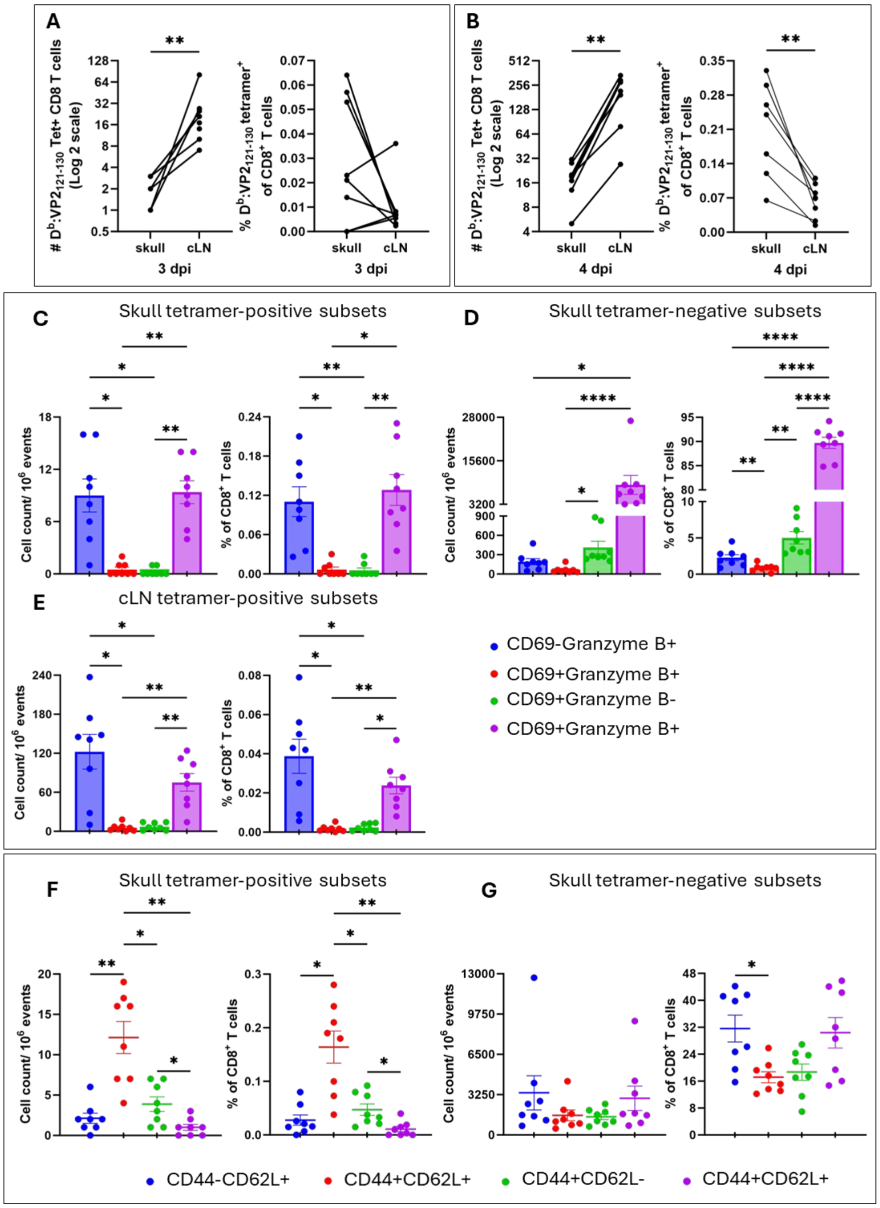

Fig. S4: Different subsets of tetramer-positive and tetramer-negative CD8 T cells exist at 4 dpi. Paired analysis of the number and frequency of virus-specific CD8 T cells between skull BM and cLN at (A) 3 and (B) 4 dpi, using paired t-test. Number and frequency of different subsets based on the coexpression of CD69 and granzyme B from (C) virus-specific CD8 T cells (tetramer positive) in skull BM, (D) tetramer negative CD8 T cells in skull BM, and (E) virus-specific CD8 T cells in cLN. Number and frequency of (F) virus-specific CD8 T cells and (G) tetramer negative CD8 T cells in skull BM based on the coexpression of CD44 and CD62L. Data normality was tested using Shapiro-Wilk test followed by repeated measure one-way ANOVA with Sidak's multiple comparison test or Friedman test with Dunn's multiple comparisons test. Individual values are plotted with mean and SEM bars. Differences are deemed significant if P value ≤ 0.05, *: $P \leq 0.05$, **: $P \leq 0.01$, ***: $P \leq 0.001$, ****: $P \leq 0.0001$.

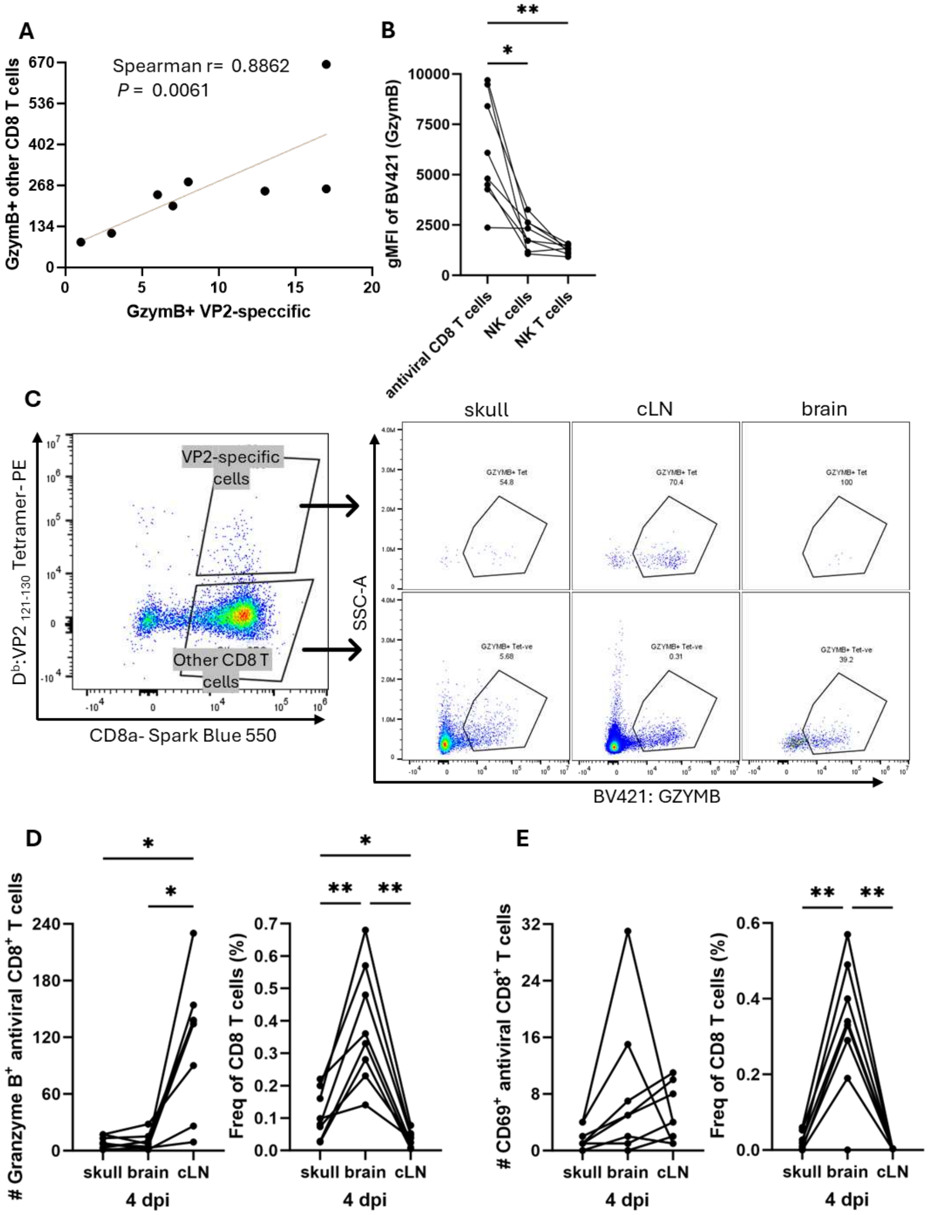

Fig. S5: Markers associated with cell activation and cytotoxicity are expressed in virus-specific CD8 T cells at 4 dpi. (A) Spearman correlation between the number of granzyme B+ tetramer positive cells and the number of granzyme B+ tetramer negative cells in skull BM at 4 dpi. (B) Protein expression of Granzyme B as indicated by gMFI of BV421 was compared between virus-specific CD8 T cells, NK cells and NK T cells in skull BM at 4 dpi, using repeated measure one-way ANOVA with Sidak's multiple comparison test. (C) Representative flow cytometry gates for granzyme B in tetramer-positive (virus-specific) and tetramer-negative CD8 T cells in skull BM, cLN, and brain. Number and frequency of (D) granzyme B+ and (E) CD69+ virus-specific CD8 T cells were compared between skull BM, brain, and cLN at 4 dpi, using repeated measure one-way ANOVA with Sidak's multiple comparison test. Differences are deemed significant if P value ≤ 0.05, *: $P \leq 0.05$, **: $P \leq 0.01$, ***: $P \leq 0.001$, ****: $P \leq 0.0001$.

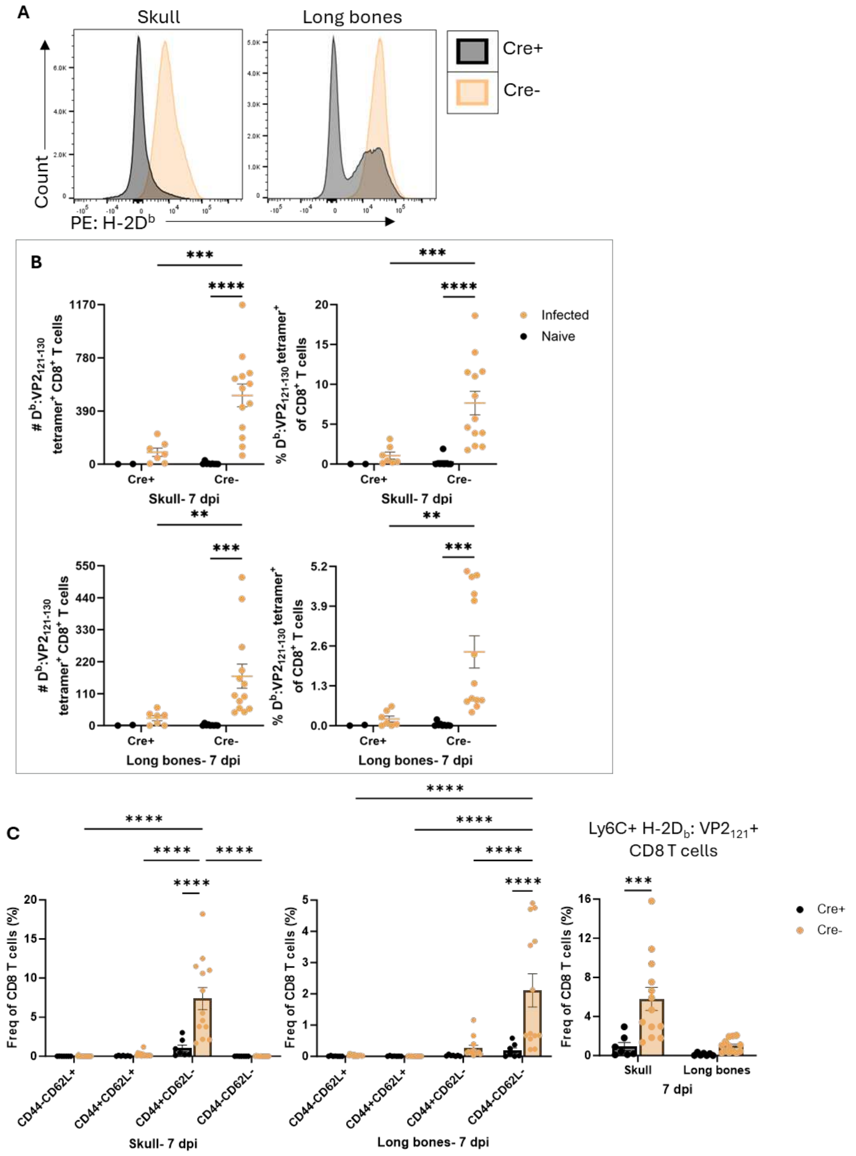

Fig. S6: Expansion of virus-specific CD8 T cells in skull BM is dependent on H-2D^b-restricted viral antigen presentation by cdh5+ APCs. (A) Representative histograms from skull BM and long bones marrow depicting H-2D^b-negative and -positive subsets in CD31+ cells in Cre+ and Cre- mice. (B) Numbers and frequencies of virus-specific CD8 T cells in transgenic mice with conditional depletion of H-2D^b in cdh5+ APCs compared to their cdh5 Cre-negative littermates in skull and peripheral BM at 7 dpi and in naïve transgenic mice. (C) Frequencies of virus-specific CD8 T cells in cdh5 Cre+ and Cre- mice based on the coexpression of CD44 and CD62L, and Ly6C expression at 7 dpi. Comparisons between groups were made using two-way ANOVA followed by Šídák's multiple comparisons and ordinary one-way ANOVA with Šídák's multiple comparisons. Individual values are plotted with mean and SEM bars. Differences are deemed significant if *P* value ≤ 0.05, *: *P* ≤ 0.05, **: *P* ≤ 0.01, ***: *P* ≤ 0.001, ****: *P* ≤ 0.0001.

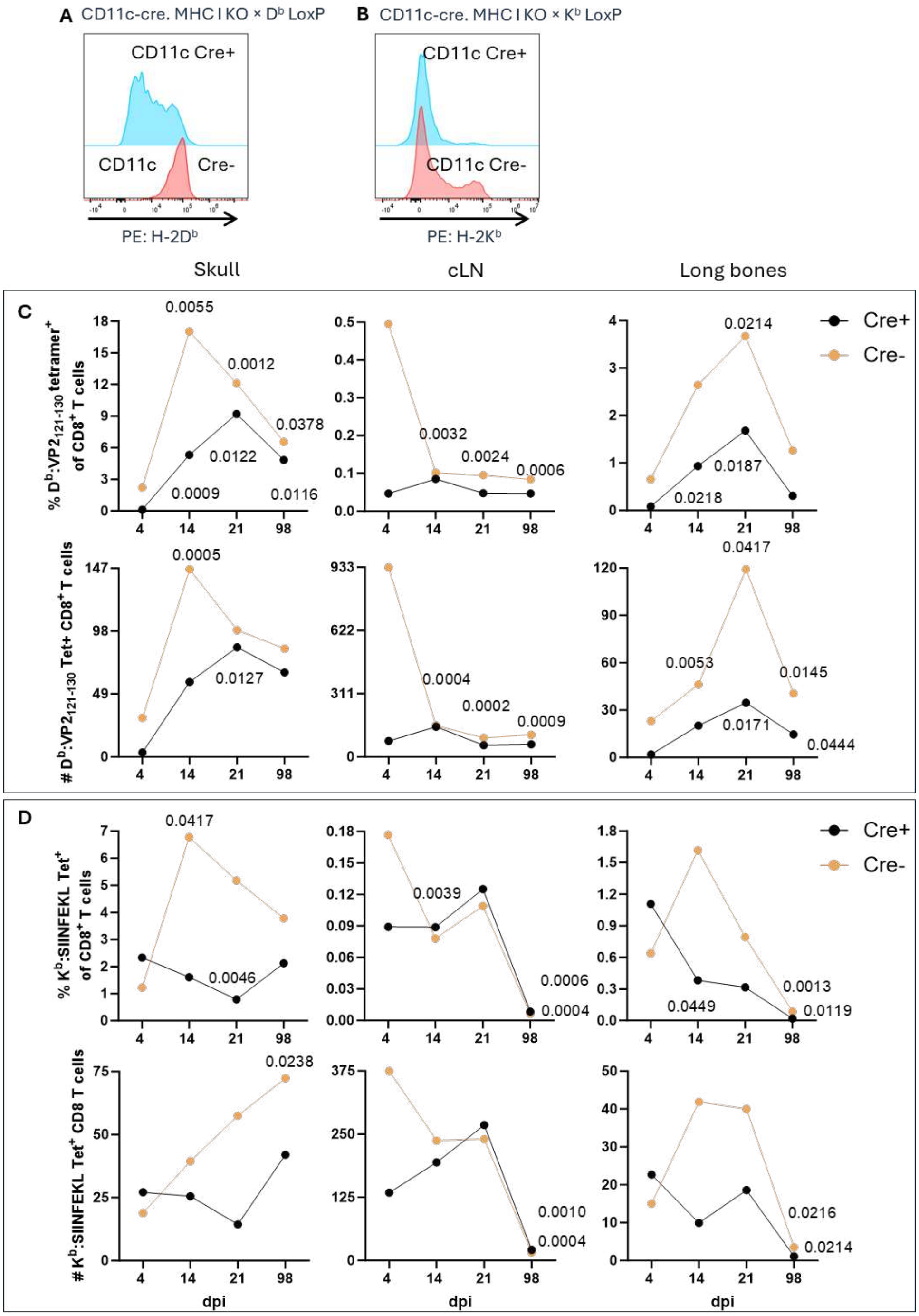

Fig. S7: Virus-specific CD8 T cells persist in skull BM during memory and chronic infection. Histogram presentation of (A) H-2D^b and (B) H-2K^b ablated expression in CD11c+ APCs in CD11c Cre+ mice in comparison to CD11c Cre- littermates. Mean frequency and number of virus-specific CD8 T cells in skull BM, cLN, and long bone marrow activated by (C) H-2D^b- and (D) H-2K^b- restricted antigen presentation in CD11c Cre+ and Cre- mice at 4, 14, 21, and 98 dpi. Each timepoint was compared to baseline at 4 dpi using two-way ANOVA with Šídák's multiple comparisons. Significantly different means compared to 4 dpi were defined with *P* values.

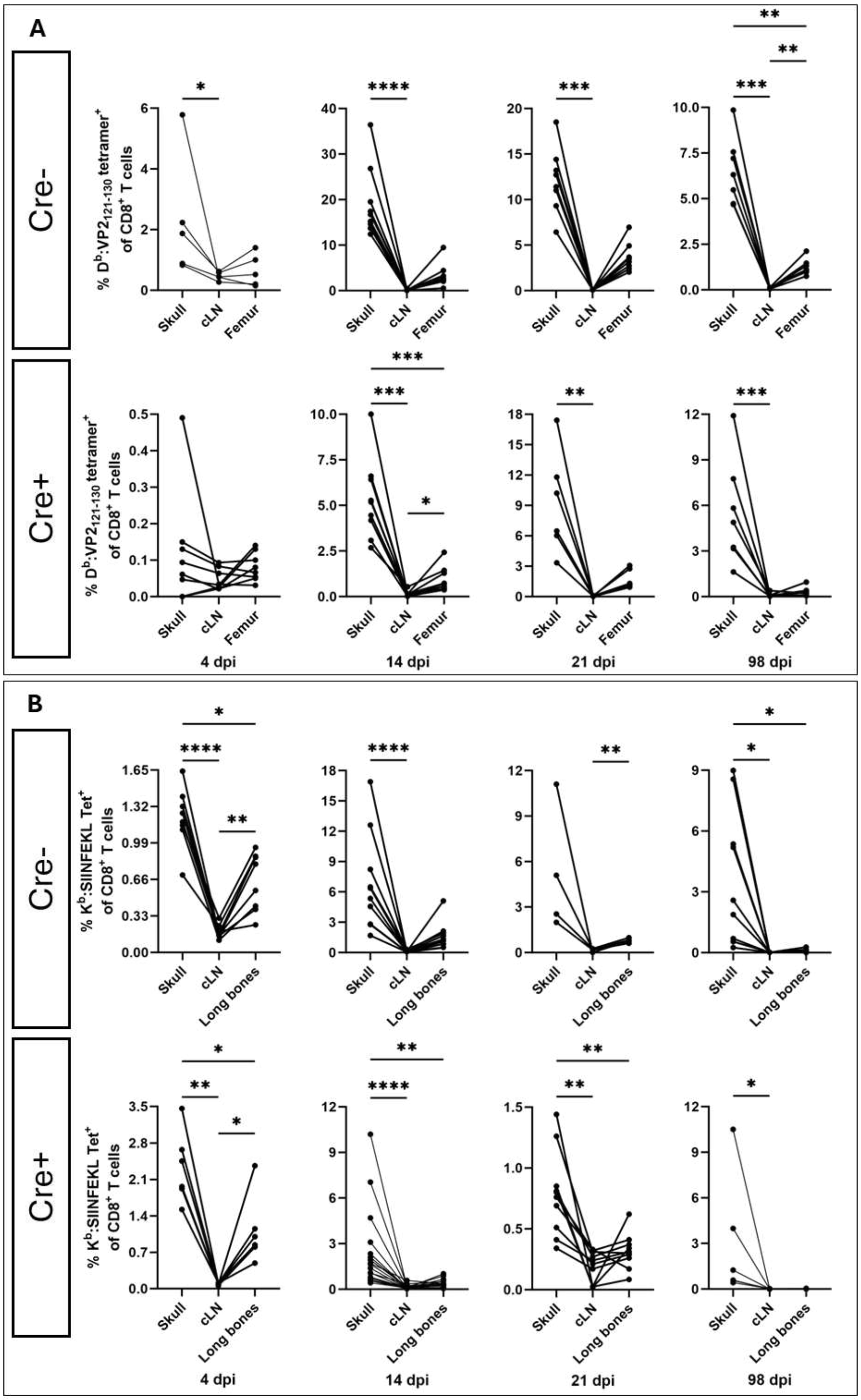

Fig. S8: Virus-specific CD8 T cells exist at considerably higher frequencies in skull BM than in cLN across different timepoints of brain infection. (A) Frequency of (A) H-2D^{b}: VP2-specific and (B) H-2K^{b}: OVA-specific CD8 T cells in skull BM, cLN, and long bone marrow in CD11c Cre- and Cre+ mice at 4, 14, 21 and 98 dpi. Comparisons were made using repeated measures one-way ANOVA followed by post-hoc Bonferroni, and deemed significant if *P* value ≤ 0.05, *: $P \leq 0.05$, **: $P \leq 0.01$, ***: $P \leq 0.001$, ****: $P \leq 0.0001$.

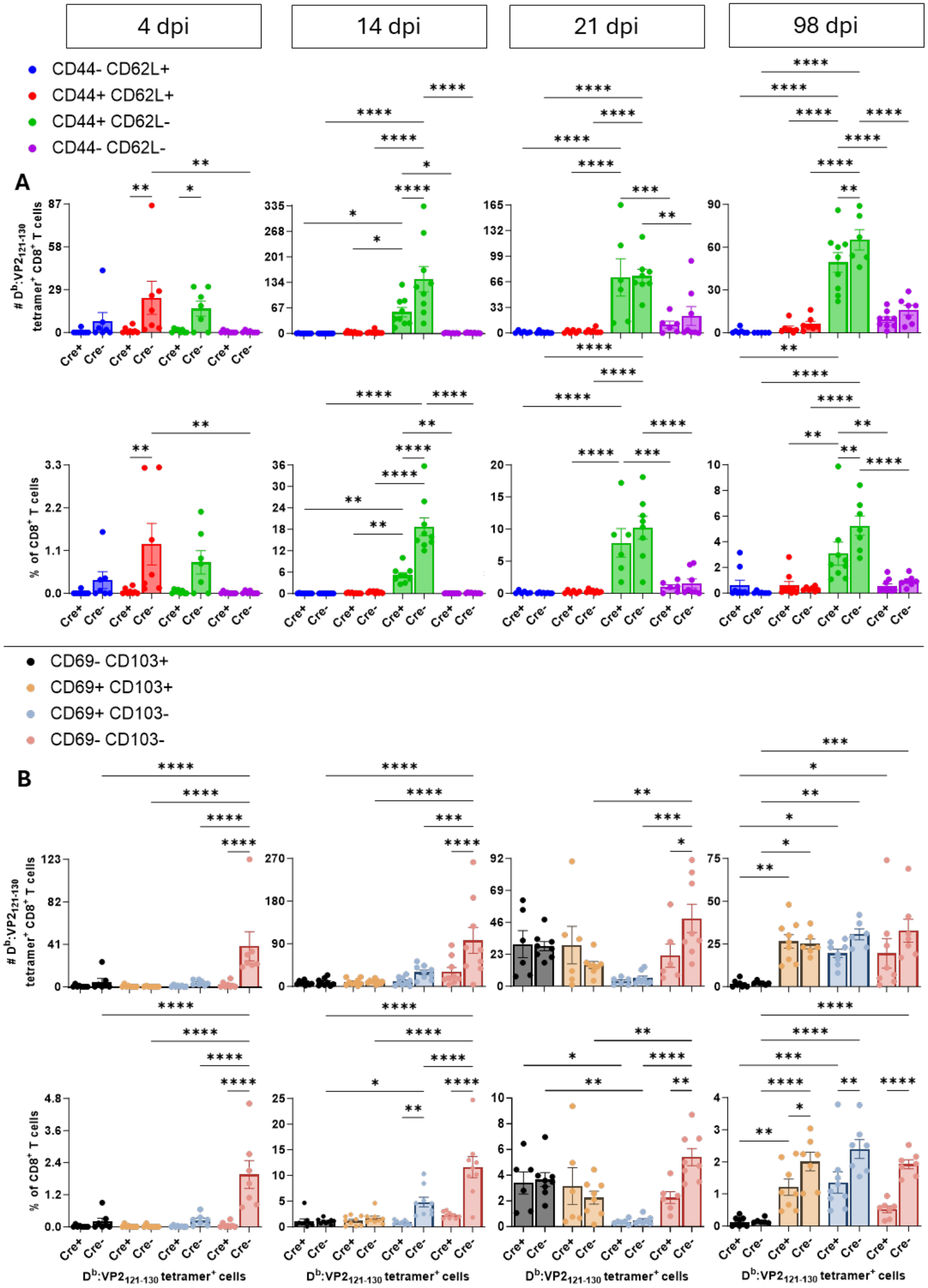
4 dpi
14 dpi
21 dpi
98 dpi
CD44- CD62L+
CD44+ CD62L+
CD44+ CD62L-
CD44- CD62L-
A
D^b:VP2121-130 tetramer+ CD8+ T cells
% of CD8+ T cells
Cre+
Cre-
CD69- CD103+
CD69+ CD103+
CD69+ CD103-
CD69- CD103-
B
D^b:VP2121-130 tetramer+ cells

Fig. S9: Conditionally deleted H-2D^b in CD11c+ APCs alters various subsets of virus-specific CD8 T cells in skull BM. Number and frequency of virus-specific CD8 T cell subsets based on the (A) coexpression of CD44 and CD62L and (B) coexpression of CD69 and CD103 in CD11c Cre+ and Cre- mice at 4, 14, 21, and 98 dpi. Data was compared using two-way ANOVA followed by Šídák's multiple comparisons and deemed significant if P value ≤ 0.05, *: $P \leq 0.05$, **: $P \leq 0.01$, ***: $P \leq 0.001$, ****: $P \leq 0.0001$. Bar graphs display individual values with mean and SEM bars.

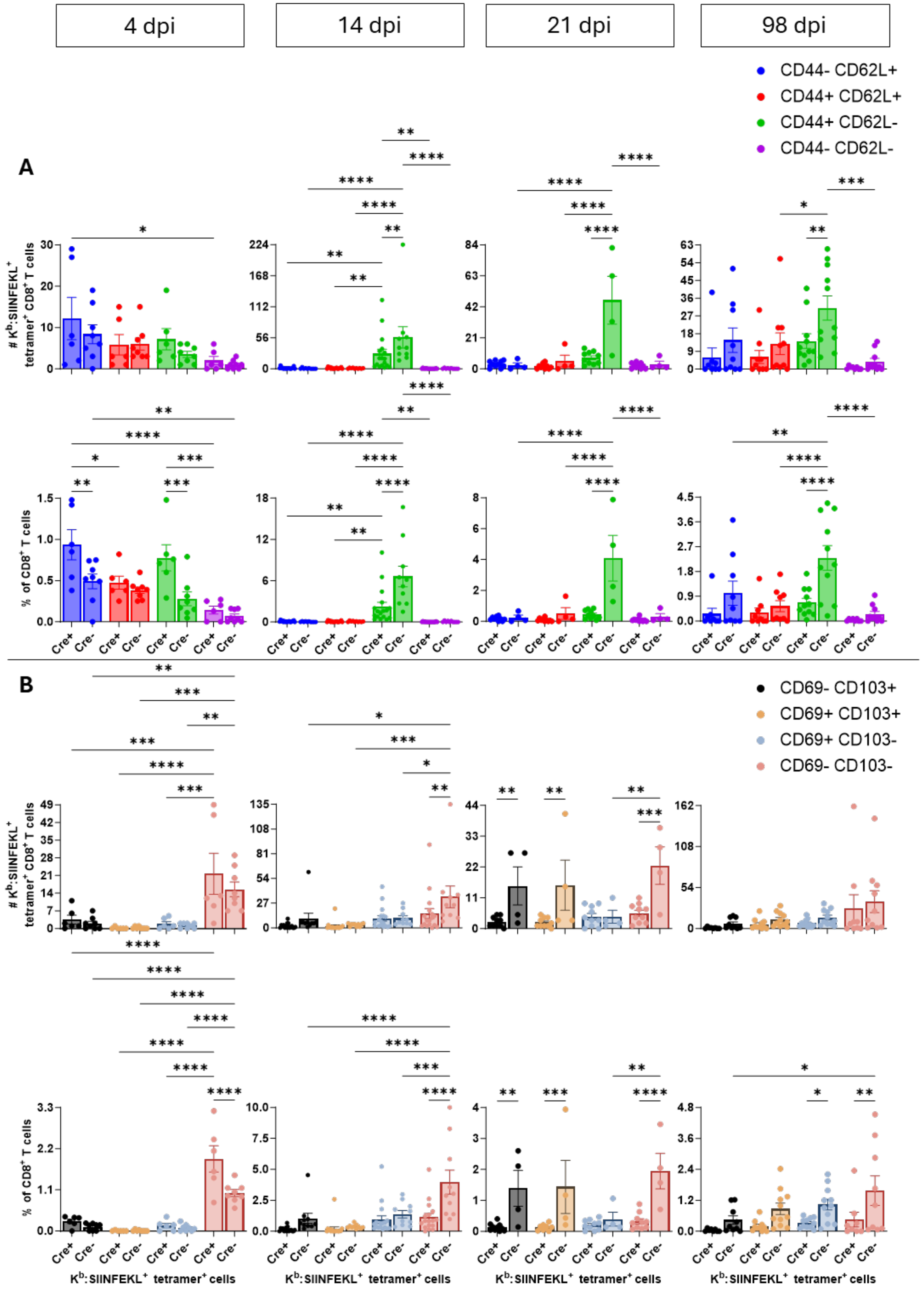
4 dpi
14 dpi
21 dpi
98 dpi
CD44- CD62L+
CD44+ CD62L+
CD44+ CD62L-
CD44- CD62L-
A
Kb:SIINFEKL+ tetramer+ CD8+ T cells
% of CD8+ T cells
Cre+
Cre-
B
CD69- CD103+
CD69+ CD103+
CD69+ CD103-
CD69- CD103-
Kb:SIINFEKL+ tetramer+ cells

Fig. S10: Conditionally deleted H-2K^b in CD11c +APCs alters various subsets of virus-specific CD8 T cells in skull BM. Number and frequency of virus-specific CD8 T cell subsets based on the (A) coexpression of CD44 and CD62L and (B) coexpression of CD69 and CD103 in CD11c Cre+ and Cre- mice at 4, 14, 21, and 98 dpi. Data was compared using two-way ANOVA followed by Šídák's multiple comparisons and deemed significant if P value ≤ 0.05, *: $P \leq 0.05$, **: $P \leq 0.01$, ***: $P \leq 0.001$, ****: $P \leq 0.0001$. Bar graphs display individual values with mean and SEM bars.

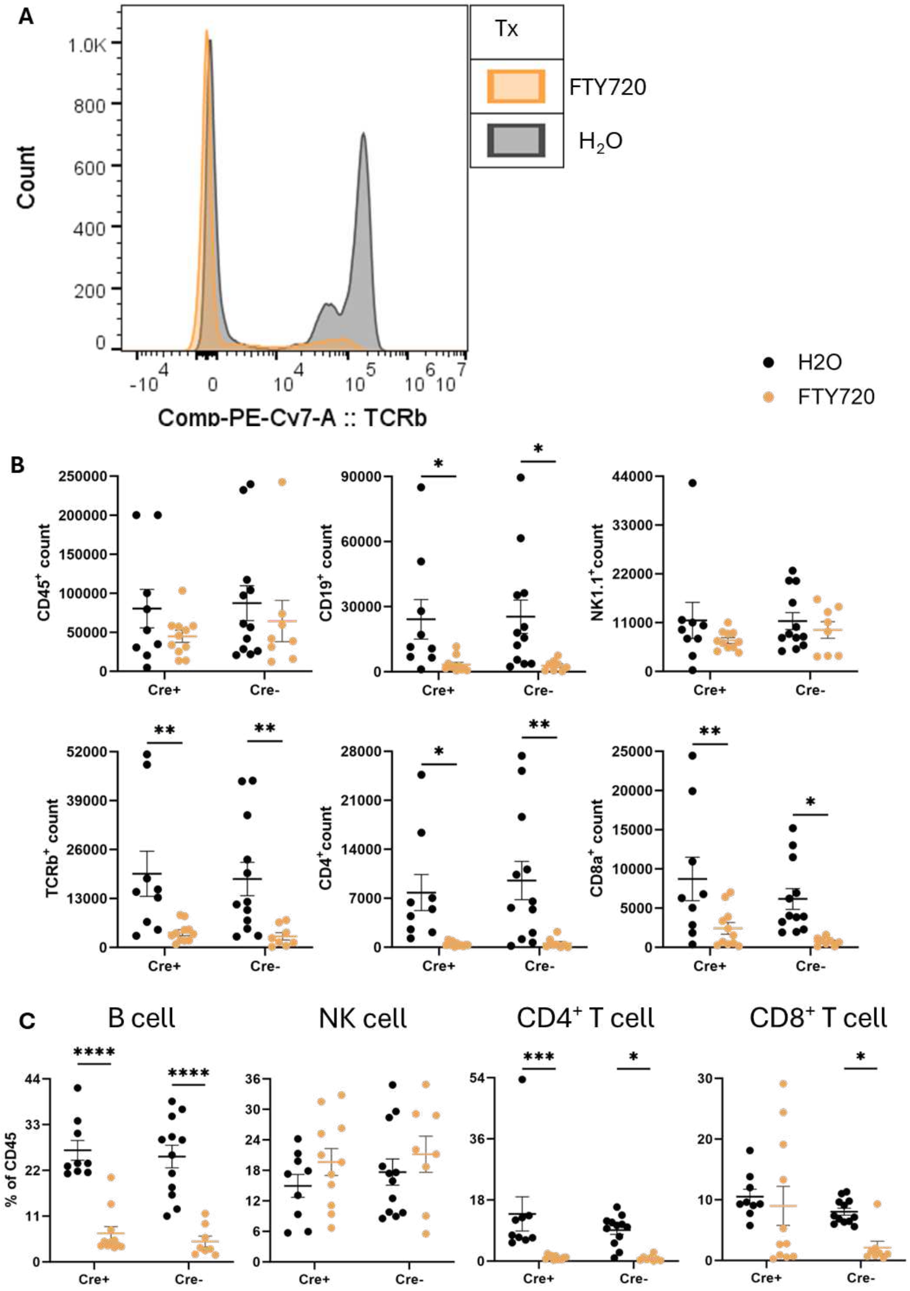

Fig. S11: FTY720 reduces the number and frequency of blood circulating lymphocytes. (A) Representative histogram depicting TCRβ+ subset in the blood following FTY720 treatment versus water (control) treatment. (B) Number and (C) frequency of various immune cells in the blood in CD11c Cre+ and Cre- mice after treatment with FTY720 or water. Groups were compared using two-way ANOVA and Šídák's multiple comparisons. Individual values are plotted with mean ± SEM. Significant differences are denoted by * if P value ≤ 0.05, *: $P \leq 0.05$, **: $P \leq 0.01$, ***: $P \leq 0.001$, ****: $P \leq 0.0001$.

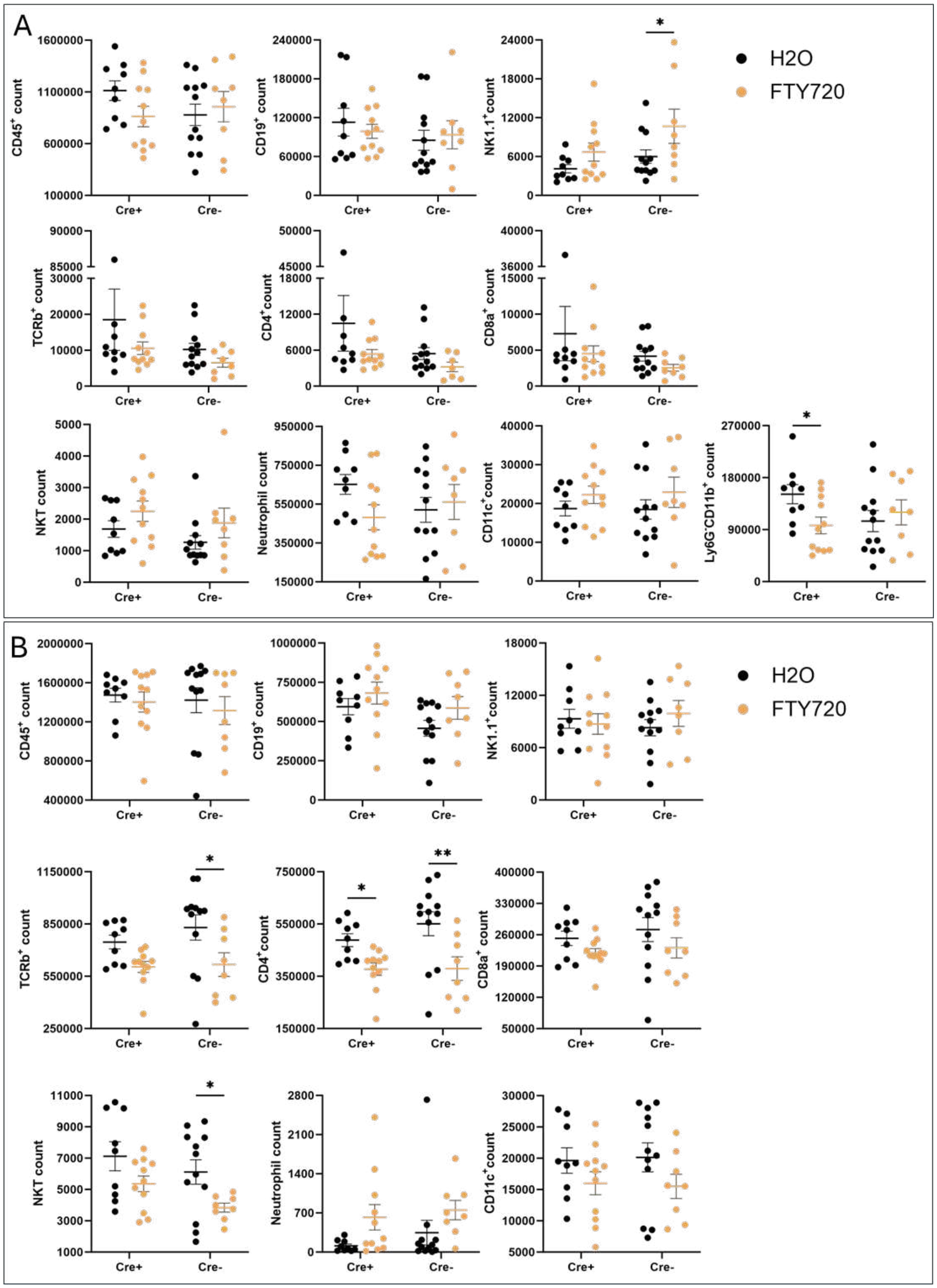

Fig. S12: Various immune cells remain numerically intact in skull BM and cLN following FTY720 treatment. Number of various immune cells in (A) skull BM and (B) cLN in CD11c Cre+ and Cre- mice following treatment with FTY720 or water. Groups were compared using two-way ANOVA and Šídák's multiple comparisons. Individual values are plotted with mean ± SEM. Significantly different groups are denoted by * if P value ≤ 0.05, *: $P \leq 0.05$, **: $P \leq 0.01$, ***: $P \leq 0.001$, ****: $P \leq 0.0001$.

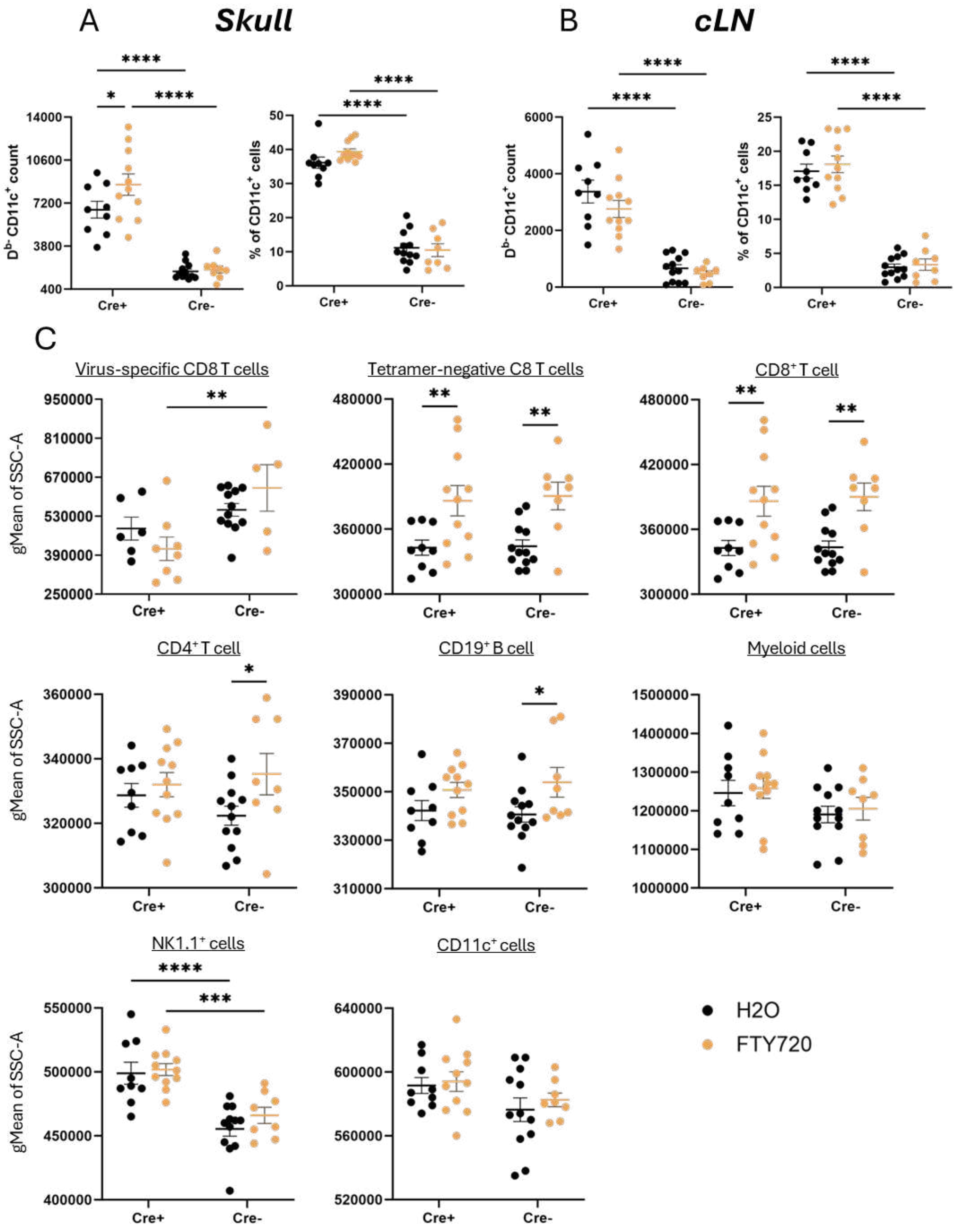

Fig. S13: FTY720 treatment impacts immune cells in skull BM differentially in CD11c Cre+ and Cre-mice. Number and frequency of H-2D^b-negative CD11c+ subset in (A) skull BM and (B) cLN in CD11c Cre+ and Cre-mice following treatment with FTY720 versus water. (C) Geometric mean of the side scatter of various immune cells as a surrogate marker for cell granularity was compared between FTY720-treated and water-treated groups in CD11c Cre+ and Cre- mice. Comparisons between groups were made using two-way ANOVA and Šídák's multiple comparisons. Individual values are plotted with mean ± SEM. Significantly different groups are denoted by * if P value ≤ 0.05, *: $P \leq 0.05$, **: $P \leq 0.01$, ***: $P \leq 0.001$, ****: $P \leq 0.0001$.

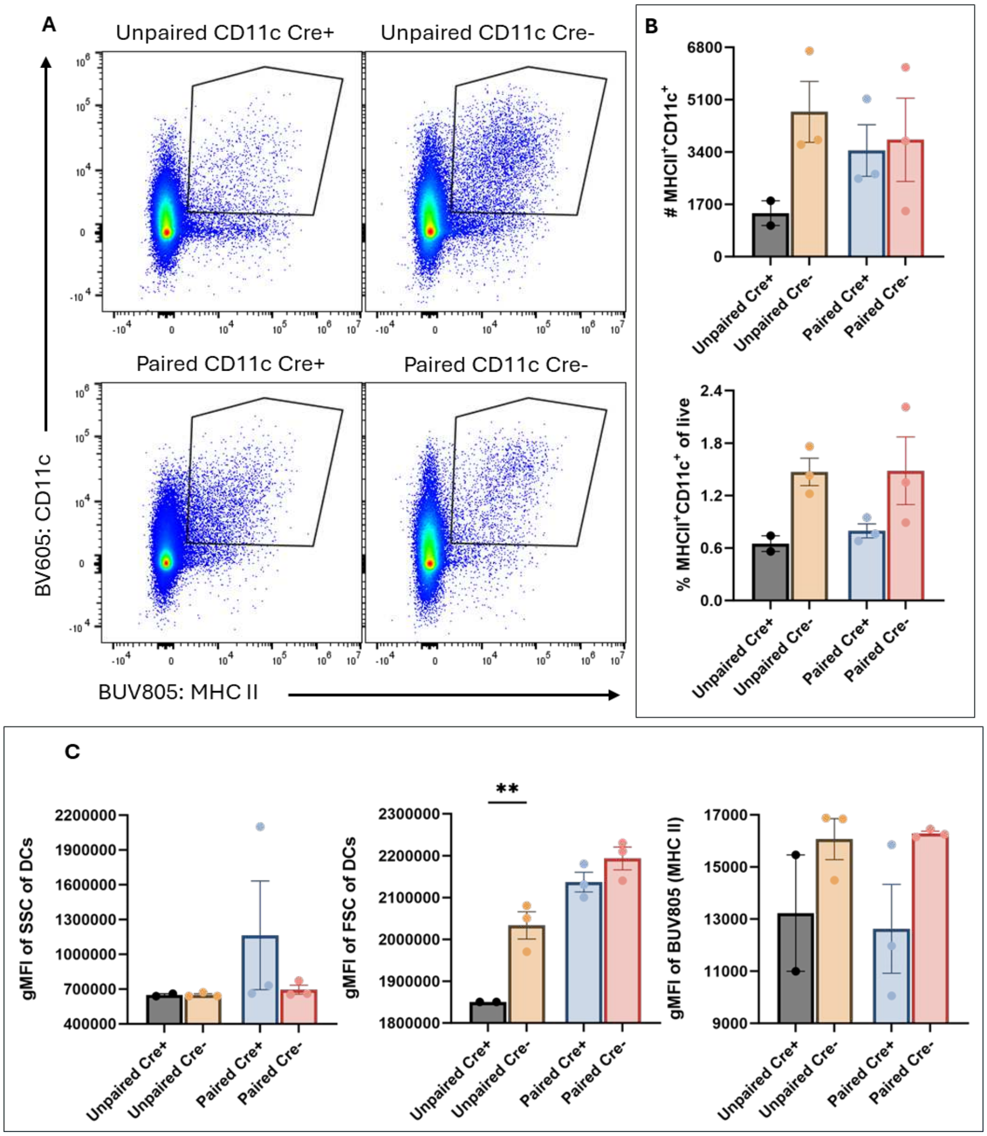

Fig. S14: Shared circulation between CD11c Cre+ and Cre- mice restores physical properties of DCs in Cre+ mice in skull BM during acute TMEV infection. (A) Representative flow gates on MHCll+CD11c+ DCs in individual CD11c Cre+ and Cre- mice with no prior shared circulation and in parabionts. (B) Number and frequency of MHCll+CD11c+ DCs in individual CD11c Cre+ and Cre- and in parabionts. (C) Geometric mean of the side scatter and forward scatter of MHCll+CD11c+ DCs and protein expression of MHC II on DCs in individual CD11c Cre+ and Cre- and in parabionts. Data was analyzed using two-way ANOVA and Šídák's multiple comparisons. Individual values are plotted with mean ± SEM. Significantly different groups are denoted by * if *P* value ≤ 0.05, *: *P* ≤ 0.05, **: *P* ≤ 0.01, ***: *P* ≤ 0.001, ****: *P* ≤ 0.0001.

Table S1: Antibodies used in this study for staining cell suspensions for flow cytometry analysis

Antibody	Clone	Cat#	Dilution
PE-CF594 α**CD45**	30-F11	Cat#: 562420, BD	1:1000
PerCP α**CD45**	30-F11	Cat#: 103130, Biolegend	1:500
PE-Cy7 α**TCRβ**	H57–597	Cat#: 60-5961-U100, Tonbo	1:100
BUV737 α**TCRβ**	H57-597	Cat#: 612821, BD	1:200
BV786 α**CD4**	RM4-5	Cat#: 563727, BD	1:50
BV510 α**CD4**	RM4-5	Cat#: 100559, Biolegend	1:50
BV570 α**CD8a**	53-6.7	Cat#: 100740, Biolegend	1:100
BV785 α**CD8a**	53-6.7	Cat # 100750, Biolegend	1:100
SparkBlue 550 α**CD8a**	53-6.7	Cat#: 100780, BioLegend	1:200
BV605 α**CD11c**	N418	Cat#: 117333, Biolegend	1:100
BV650 α**CD11c**	HL-3	Cat#: 564079, BD	1:100
PE α**H-2D**^{b}	28-14-8	Cat#: A15443, Invitrogen	1:50
PerCP/Cy5.5 α**H-2D**^{b}	KH95	Cat#: 111517, Biolegend	1:50
PE α**H-2K**^{b}	AF6-88.5	Cat#: 553570, BD	1:50
BB700 α**H-2K**^{b}	AF6-88.5	Cat #: 4061114, BD	1:50
PE-Cy7 α**CD31**	390	Cat#: 25-0311-82, Invitrogen	1:100
BV750 α**MHC class I**	28-8-6	Cat#: 746869, BD	1:100
BV786 α**MHC class I**	M1/42	Cat#: 749706, BD	1:500
BUV395 α**F4/80**	T45-2342	Cat#: 565614, BD	1:200
BUV615 α**NK1.1**	PK136	Cat#: 751111, BD	1:100
BUV805 α**MHC Class II**	M5114.15.2	Cat#: 748844, BD	1:200
PE-Cy5 α**CD11b**	M1/70	Cat#: 55–0112-U100, Tonbo	1:1000
BV711 α**Ly6G**	1A8	Cat#127643, Biolegend	1:200
APC-Fire 810 α**CD19**	6D5	Cat#: 115578, Biolegend	1:100
BUV737 α**CD21/35**	7G6	Cat#: 612810, BD	1:500
